# Environmental context alters plant–soil feedback effects on plant coexistence

**DOI:** 10.1002/ecy.70170

**Published:** 2025-08-06

**Authors:** Jeremy A. Collings, Lauren G. Shoemaker, Jeffrey M. Diez

**Affiliations:** ^1^ Department of Biology University of Oregon Eugene Oregon USA; ^2^ Botany Department University of Wyoming Laramie Wyoming USA

**Keywords:** coexistence, context‐dependency, fitness ratio, mutualisms, niche differences, pathogens, plant–soil feedbacks, stabilizing mechanisms

## Abstract

Plant–soil feedbacks are thought to mediate outcomes of plant competition through microbially driven positive or negative feedback loops. Plant–microbe interactions are known to depend on the underlying environmental context, yet most efforts to understand how plant–soil feedbacks mediate species coexistence have not considered these context dependencies. Here, we use modern coexistence theory to assess how this environmental context‐dependence of plant–microbe interactions might influence plant coexistence, and through which species interactions environmental context‐dependencies are most likely to modify coexistence outcomes. First, we found that the component species interactions in a plant–soil feedback model vary in their relative effects, with microbes impacting fitness ratios more so than direct plant–plant interactions. We also found that linear effects of the environment on plant–soil feedbacks result in nonlinear changes in niche differences and fitness ratios, which can result in altered predictions for coexistence, shifting species pairs from regions of coexistence to competitive exclusion or even priority effects. Finally, we extended our model to simulate how environmental dependence of plant–soil feedbacks in a well‐studied invasive species, garlic mustard (*Alliaria petiolata*), may help explain invasion dynamics across environmental regions. We also discuss paths forward in empirically parameterizing context‐dependent coexistence models using greenhouse experiments. This work builds on recent efforts to integrate plant–soil feedback theory with modern coexistence theory, suggesting that context‐dependencies of plant–microbe interactions may more strongly alter fitness ratios compared to plant–plant interactions. Our results highlight how context‐dependent plant–soil feedbacks may help explain spatial and temporal variation in plant community dynamics and suggest that the underlying mechanism of these dynamics depends on the relative sensitivity of particular species interactions to the environmental gradient.

## INTRODUCTION

A central goal in community ecology is to understand the mechanisms that maintain species coexistence and promote ecological diversity, for example, leading to different spatial patterns of species' contemporary distributions (Usinowicz & Levine, 2018) or temporal variability in dominance (Hallett et al., 2019). Understanding how coexistence is mediated by environmental conditions—and their inherent variability—is crucial for predicting how global change might alter these patterns of distributions and range limits, species interactions, and coexistence (Valladares et al., 2015). Modern coexistence theory (MCT, Chesson, 2000) has become a powerful conceptual framework for investigating the underlying mechanisms maintaining species diversity and has highlighted how differences among species may either promote coexistence or lead to competitive exclusion. This framework has recently been integrated with plant–soil feedback (PSF) theory (as originally formulated by Bever et al., 1997) to predict how plant–microbe interactions may influence the outcomes of plant competition (Kandlikar, 2024; Ke & Wan, 2020).

Efforts to study PSFs using MCT have, however, neglected an important finding from plant–microbe research: that the strength and even direction of plant–microbe interactions can change depending on environmental conditions (Hahn et al., 2018; Nuske et al., 2021; Van Nuland et al., 2023). PSFs may exhibit environmental context‐dependency either due to changes in the microbial community across environmental gradients (Francioli et al., 2018; Pérez‐Jaramillo et al., 2019), or shifts in the function of particular plant–microbe interactions. The physiology of plant and/or microbial symbionts is known to depend on environmental conditions, shifting pairwise interaction along a mutualism–parasitism gradient (Johnson et al., 1997; Rogalski et al., 2021). Accounting for these context‐dependencies is among the foremost challenges in applied PSF research (Smith‐Ramesh & Reynolds, 2017; van der Putten et al., 2016), and although many empirical studies have highlighted its importance for PSFs (Dudenhöffer et al., 2022; Manning et al., 2008; Smith & Reynolds, 2015), context‐dependent PSFs have not been incorporated into theory, making it unclear how much specific empirical results may generalize.

MCT is especially well suited to address context‐dependent PSFs as it provides a framework for disentangling the effects of the environment and competitive interactions on species' fitness and niche differences (Godoy & Levine, 2014; Van Dyke et al., 2022; Wainwright et al., 2019). The general approach for using MCT to predict the outcome of competition involves parameterizing population models to calculate the effects of density‐independent growth, intraspecific, and interspecific competition on species realized fitness (Hallett et al., 2019; Kraft et al., 2015). These competition coefficients can then be used to calculate the niche differences and fitness ratios between species, and predict whether coexistence, competitive exclusion, or priority effects will occur (Chesson, 2000; Grainger et al., 2019). MCT provides the conceptual and mathematical framework to then determine whether any process (such as PSF) that affects these niche differences (stabilization) and/or fitness ratios (equalization) will shift competitive interactions enough to alter coexistence outcomes.

Recent studies have demonstrated the value of modeling PSFs using the MCT framework. For example, Kandlikar et al. (2019) use an MCT framework to explore traditional models of PSFs and found that explicitly including PSF effects on fitness ratios, in addition to niche differences, can alter predictions of PSF‐mediated competition compared to traditional models. An empirical application of this work found that, in fact, microbial effects on fitness ratios rather than niche differences drove the outcomes of PSFs (Kandlikar et al., 2021). Ke and Wan (2020) similarly developed demographic models of PSF‐mediated plant population and microbial community growth to predict coexistence outcomes between competing plant populations, finding that soil microbes and plant‐competitive interactions jointly determine coexistence outcomes. Although these studies have not incorporated the context‐dependence of PSFs, MCT is well suited to do so because of its ability to assess the role of environmental effects in mediating species interactions (Hart & Marshall, 2013; Van Dyke et al., 2022; Wainwright et al., 2019). For example, MCT can be used to compare coexistence outcomes between sites along spatial gradients or between current and projected environmental conditions.

Another benefit of using MCT to study context‐dependent PSFs is that this framework can include detailed mechanisms of how species interact while remaining analytically tractable. As a consequence, the relative importance of different interactions for driving coexistence can be assessed. For example, understanding how an abiotic variable affects PSFs and plant coexistence depends on quantifying the effect of the abiotic variable on specific species interactions and the effect of these species interactions on niche differences and fitness ratios. Although empirically quantifying all of the species interactions within a complex PSF network may be difficult (Shang et al., 2017), sensitivity analyses may be used to assess which interactions are most important for niche differences and fitness ratios (Cariboni et al., 2007). The sensitivities of niche differences and fitness ratios can help identify which species interactions and which abiotic gradients should be the focus of empirical field and greenhouse studies.

In this study, we develop a set of models to predict species coexistence outcomes that allow for environmental context‐dependency of plant–microbe interactions. We first use these models to predict how context‐dependency of PSFs may impact species coexistence in a general theoretical model, highlighting the flexibility of this approach to accommodate different levels of microbial community complexity and different microbial guilds, including pathogens, mutualists, and decomposers. We then conduct a set of sensitivity analyses on the microbe‐dependent plant niche difference and fitness ratio equations to compare how different environmental dependencies of particular species interactions vary in their influence on niche versus fitness differences. Finally, we adapt our models to a case study of the invasion dynamics of garlic mustard (*Alliaria petiolata*), showing how plant–microbe interactions can mediate potential range expansion and coexistence patterns.

## METHOD OVERVIEW

### General theoretical model

**FIGURE 1:**
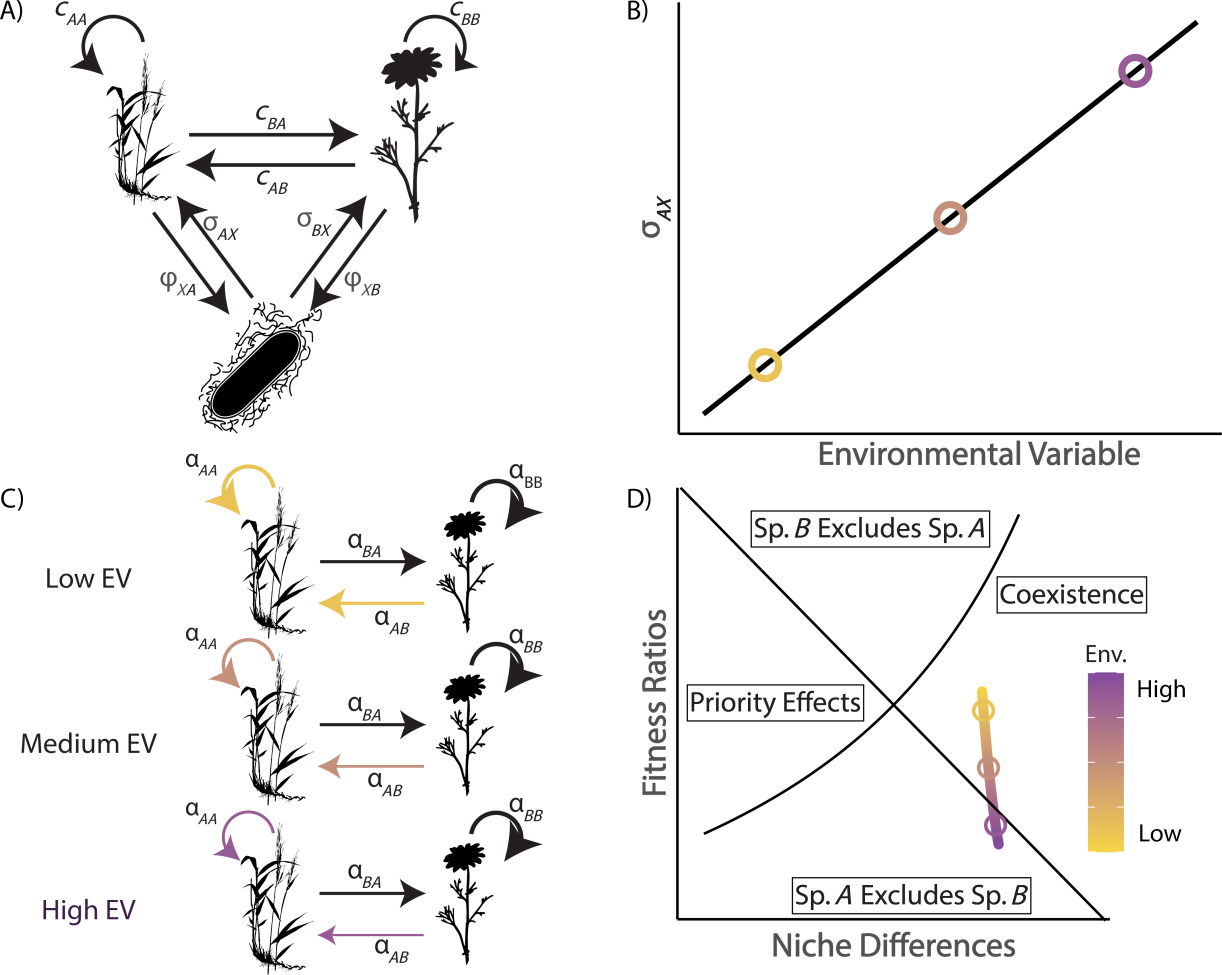
Overview of our approach to modeling environmentally context‐dependent plant–soil feedback (PSF)‐mediated coexistence. We first constructed a taxon‐explicit model of a plant–soil feedback (A) in which any number of microbial taxa may have taxon‐specific interactions with the two competing plant species. Plants compete with one another directly through the microbe‐independent competition coefficients (c) and indirectly through the cultivation of soil microbes (ϕ) and the consequential effect of these microbes on each plant species (σ). We define a function (B) that scales a parameter of interest ($\sigma_{\mathrm{AX}}$ in this example, though other parameter scaling combinations are explore elsewhere in the text) across an environmental gradient. Colored circles indicate discrete environmental contexts to be examined in (C) and (D). The downstream effect of scaling this particular parameter is shown in (C) where overall competition coefficients (α) vary depending on where the species interaction is taking place along the environmental gradient. Finally, these shifts in competition coefficients lead to shifts in niche differences and fitness ratios, (D) that might alter predicted coexistence outcomes. Here, the environmental context‐dependency of these plant–microbe interactions acts as an equalizing mechanism, reducing fitness ratios at low values of the environmental variable (EV) enough to maintain stable coexistence. Organism silhouettes retrieved from PhyloPic and modified. Species *A* uses *Brachyelytrum erectum* by T. Michael Keesey under a Public Domain Mark 1.0 license; species *B* uses *Chamaemelum fuscatum* by David García‐Callejas under a CC0 1.0 Universal Public Domain Dedication license; microbial taxon *X* uses *Sonnebornia yantaiensis* by Matt Crook under an Attribution‐ShareAlike 3.0 Unported license (https://creativecommons.org/licenses/by‐sa/3.0/).

Our models extend the approach formulated by Ke and Wan (2020), with two important adaptations. First, we developed a taxon‐specific model of PSFs instead of treating the microbial community as a single generic entity (Figure 1A). Second, we allowed parameters of the models to vary depending on environmental conditions. These two extensions allowed us to incorporate more biological realism and discern the specific contributions of pathogenic or mutualist taxa, as well as the role of environmental variation for shaping coexistence outcomes. We use this model structure with phenomenological interaction coefficients because this approach is similar to other approaches that treat species interactions as dependent on environmental conditions (Hallett et al., 2019; Van Dyke et al., 2022; Wainwright et al., 2019). However, when species interactions are dependent on the abundance of consumable resources, system dynamics may be determined by more complicated feedbacks between plants, microbes, and resources, which may be captured better with more mechanistic resource‐consumption models.

In this formulation, competing plant population growth was modeled for each plant species A and B:

(1) $$\frac{{\mathrm{dN}}_{A}}{\mathrm{dt}}=r_{A}N_{A}\left(1+c_{\mathrm{AA}}N_{A}+c_{\mathrm{AB}}N_{B}+\sigma_{\mathrm{AX}}S_{X}\right)$$

(2) $$\frac{{\mathrm{dN}}_{B}}{\mathrm{dt}}=r_{B}N_{B}\left(1+c_{\mathrm{BB}}N_{B}+c_{\mathrm{BA}}N_{B}+\sigma_{\mathrm{BX}}S_{X}\right)$$

where $N_{i}$ is the population sizes of plant species A when i=A and species B when i=B, respectively. Parameter $r_{i}$ is the intrinsic growth rate, $c_{\mathrm{ii}}$ denotes the intraspecific competitive effect, $c_{\mathrm{ij}}$ denotes the competitive effect of plant species j on species i, and $\sigma_{\mathrm{iX}}$ is the effect of a microbial taxon X on plant population i. $S_{X}$ is the population size of microbial taxon X, and its growth was modeled using the logistic equation:

(3) $$\frac{{\mathrm{dS}}_{X}}{\mathrm{dt}}=g_{X}S_{X}\left(1-\frac{S_{X}}{k_{X}}\right)$$

where $g_{X}$ is the intrinsic growth rate of the microbe X. To reflect the expectation that microbe abundance is strongly affected by the plants, the carrying capacity of the microbe, $k_{X}$, was defined as

(4) $$k_{X}=\phi_{\mathrm{XA}}N_{A}+\phi_{\mathrm{XB}}N_{B}$$

where $\phi_{\mathrm{Xi}}$ is the rate by which each plant i cultivates the microbe X. This adaptation of the original models proposed by Ke and Wan (2020) allowed flexibility to include any number of microbial taxa as well as the ability for each taxon to have either generalist or species‐specific $\sigma_{\mathrm{iX}}$ and $\phi_{\mathrm{Xi}}$ terms.

To incorporate environmental context‐dependence of PSF, each model parameter above (i.e., $c_{\mathrm{ij}}$, $\sigma_{\mathrm{iX}}$, or $\phi_{\mathrm{Xi}}$ terms) could vary with environmental conditions according to a scaling function (Figure 1B). Here, we focused on the direct effect of microbial taxa on plant population size ($\sigma_{\mathrm{iX}}$) because of its central importance in PSF research (Bever, 2003; Bever et al., 1997; Caruso & Rillig, 2022), and conducted a sensitivity analysis (described below) to determine the importance of each parameter on overall outcomes. We also assumed a linear effect of environmental conditions on these parameters, but explored the implications of nonlinear relationships (Appendix S1). Thus, a generic structure for these linear scaling functions of, for example parameter $\sigma_{\mathrm{iX}}$, where v is an environmental variable, is:

(5) $$\sigma_{\mathrm{iX}}={\hat{\sigma }}_{0,\mathrm{iX}}+v\times {\hat{\sigma }}_{1,\mathrm{iX}}$$

where the observed value of the microbial effect on a plant species, $\sigma_{\mathrm{iX}}$, is a function of the intercept of the linear scaling function, ${\hat{\sigma }}_{0,\mathrm{iX}}$, the value of the environmental variable, v, and the sensitivity of $\sigma_{\mathrm{iX}}$ to the v, ${\hat{\sigma }}_{1,\mathrm{iX}}$. The hat denotes intermediate parameters that alter the net microbial‐plant interactive effect.

### Calculating coexistence

Similar to Ke and Wan (2020) and consistent with the two‐species Lotka‐Volterra formalization (Chesson, 2013), we calculated the two axes of coexistence—fitness ratios and niche differences—as a function of the interspecific and intraspecific competition coefficients ($\alpha_{\mathrm{ij}}$). Using the Lotka‐Volterra model structure defined by Ke and Wan (2020), we calculate $\alpha_{\mathrm{ij}}$ values as

(6) $$\alpha_{\mathrm{ij}}=c_{\mathrm{ij}}+\sum_{X=1}^{n}\;\sigma_{\mathrm{iX}}\phi_{\mathrm{Xj}}$$

where i and j can each be either plant A or B and X are the microbial taxa.

Using these $\alpha_{\mathrm{ij}}$ values, we calculated fitness ratios as:

(7) $$\frac{f_{B}}{f_{A}}=\sqrt{\frac{\alpha_{\mathrm{AA}}\alpha_{\mathrm{AB}}}{\alpha_{\mathrm{BB}}\alpha_{\mathrm{BA}}}}$$

We calculated niche overlap (ρ) as

(8) $$\rho =\sqrt{\frac{\alpha_{\mathrm{AB}}\alpha_{\mathrm{BA}}}{\alpha_{\mathrm{AA}}\alpha_{\mathrm{BB}}}}$$

and niche differences were then defined as 1−ρ. As we have calculated them, the fitness ratios measure the relative difference between the overall competition experienced by the two species, and the niche differences measure the extent to which the interaction is stabilized by negative frequency dependency. Together, fitness ratios and niche differences can be used to evaluate the outcome of competition according to the predictions made by MCT. Given that the environment alters $\sigma_{\mathrm{iX}}$, it also modifies competition coefficients $\alpha_{\mathrm{ij}}$ and both niche differences and fitness ratios between plant species (Figure 1C,D). These metrics depend on the exact structure of the bipartite plant–microbe network, which can vary in microbial taxonomic richness and specificity of plant–microbe interactions. We explore multiple plant–microbe interaction structures in this paper, and an overview of the model structures referenced in the main text can be found in Appendix S2.

## RESULTS AND CASE STUDIES

### Theory simulations

#### Methods

We first conducted a set of theoretical simulations, with varying effects of microbes and complexity of microbial communities to test how interactions between microbes and the environment influence plant coexistence. In each of the simulations, we began with microbe‐independent competition coefficients that allowed the competing species to stably coexist: $c_{\mathrm{AA}}=-0.06$, $c_{\mathrm{BA}}=-0.05$, $c_{\mathrm{BB}}=-0.075$, and $c_{\mathrm{AB}}=-0.06$. For each set of simulations, we calculated niche differences and fitness ratios to predict coexistence, competitive exclusion, and priority effects outcomes. Because we are particularly interested in the scaling of species interaction terms, we assumed that plant species have the same intrinsic growth rates. Because of this assumption, the exact value of the plant intrinsic growth rate does not influence the results of our analyses but would alter the exact equilibrium abundances of species. Therefore, we do not specify values for intrinsic growth rates.

To first simply ask how the presence of microbes may influence plant coexistence, we simulated the competitive dynamics between these two plant species under a constant set of environmental conditions (no scaling function) and three distinct microbial parameterizations that represent three alternative systems: sterile ($\alpha_{\mathrm{ij}}=c_{\mathrm{ij}}$; ThSi I), a single mutualist associating with species *A* ($\sigma_{\mathrm{AX}}=0.002$, $\phi_{\mathrm{XA}}=10$; ThSi II), and an additional mutualist taxon associating with species *B* ($\sigma_{\mathrm{BZ}}=0.0025$, $\phi_{\mathrm{ZB}}=10$; ThSi III).

To explore how an environmental gradient can affect plant coexistence in the absence of microbially mediated context‐dependence, we then used the same baseline parameterization in sterile conditions to simulate competitive dynamics along an environmental gradient by scaling the microbe‐independent competition coefficients. We defined the scaling parameters such that species *A* experienced less competitive effects with higher values of the environmental variable (as in the case of a species limited by a particular resource). Thus, we defined the scaling parameters for the slope of competition across environmental gradient v as ${\hat{c}}_{1,\mathrm{AA}}=0.0025$, ${\hat{c}}_{1,\mathrm{BA}}=0.001$, ${\hat{c}}_{1,\mathrm{BB}}=0.001$, and ${\hat{c}}_{1,\mathrm{AB}}=0.0015$; intercepts are defined according to the sterile conditions above (${\hat{c}}_{0,\mathrm{AA}}=-0.06$, ${\hat{c}}_{0,\mathrm{BA}}=-0.05$, ${\hat{c}}_{0,\mathrm{BB}}=-0.075$, and ${\hat{c}}_{0,\mathrm{AB}}=-0.06$).

Finally, we parameterized our models to explore how microbial symbionts may indirectly mediate plant coexistence along an environmental gradient. Thus, we defined linear functions to scale both mutualists' effects on their respective plant symbionts ($\sigma_{\mathrm{iX}}$) in the ThSi III model by an environmental variable such that both mutualists became less beneficial, and even became pathogenic, as the value of the environmental variable increased (but at different rates, with ${\hat{\sigma }}_{1,\mathrm{AX}}=-0.00015$ and ${\hat{\sigma }}_{1,\mathrm{BZ}}=-0.0003$). Intercepts are defined as above, with ${\hat{\sigma }}_{0,\mathrm{AX}}=0.002$ and ${\hat{\sigma }}_{0,\mathrm{BZ}}=0.0025$.

#### Results

**FIGURE 2:**
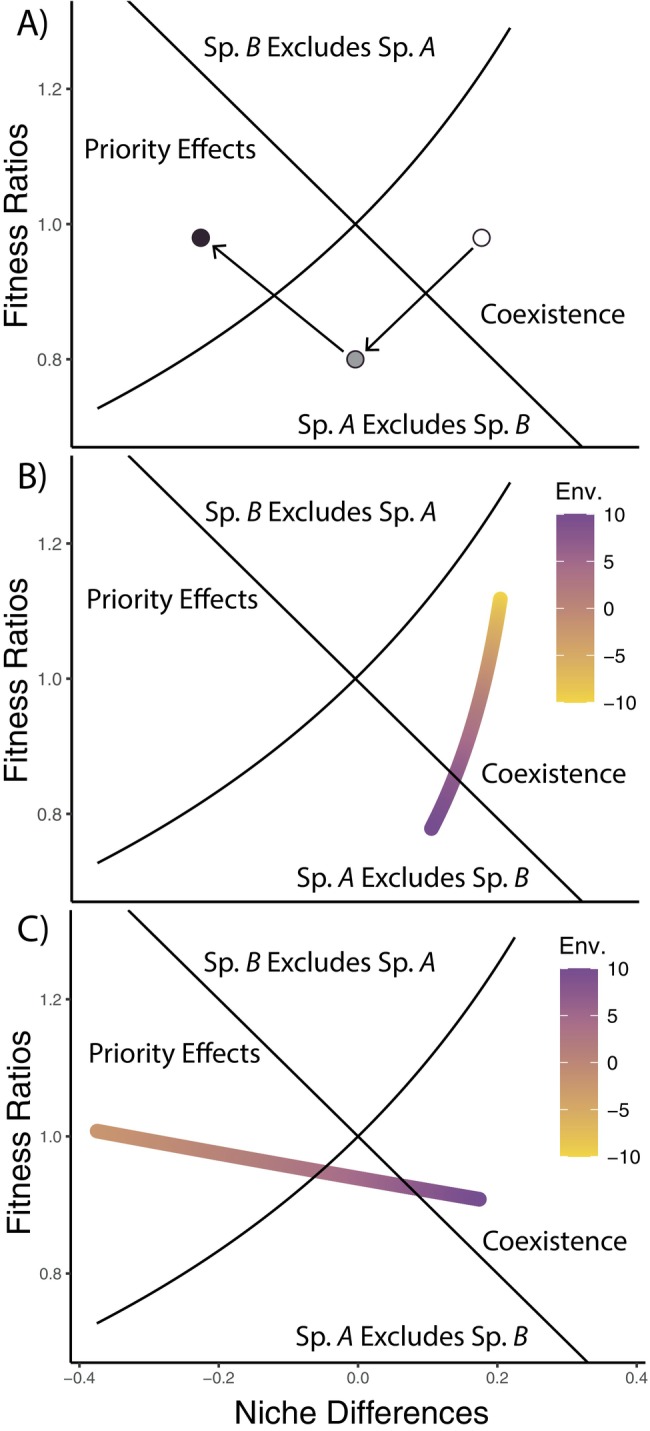
Synthesis of modeled relationships between soil microbes, environmental variables, and plant competition outcomes. (A) Simulated competition outcomes under sterile conditions (open circle) and two live conditions independent of environmental variability. Live conditions include the sequential addition of a species‐specific mutualist for sp. A (gray circle) and for sp. B (black circle). Arrows represent how the addition of microbes might alter niche differences and/or fitness ratios between competing species, thereby altering the predicted outcome of competition. (B) Simulated competition outcomes in sterile conditions along an environmental gradient independent of microbes. The circle around the first colored point indicates the point in coexistence space in live conditions when the value along the environmental gradient is 0. (C) Simulated competition outcomes live conditions (colored points) along an environmental gradient. The circle around the first colored point indicates the point in coexistence space in live conditions when the value along the environmental gradient is 0. The environmental variable affects plant competition indirectly through linear scaling of σ terms.

Introducing a mutualist associated with species *A* resulted in a loss of coexistence due to reduced intraspecific competitive effects for species *A* (Figure 2A, gray circle). While one species possessing a mutualist allows this species to outcompete the other, when both species have species‐specific mutualists (Figure 2A, black circle) decreased niche differences destabilize coexistence, thus resulting in priority effects, where competitive exclusion is based on species arrival order. These simulations demonstrate the possible destabilizing effects of species‐specific mutualists.

We also assessed the role of environmental variation, independent of microbial effects, in influencing plant species coexistence through fluctuation‐independent mechanisms of coexistence (Figure 2B). Scaling competition coefficients linearly by the environmental variable yielded different coexistence outcomes along an environmental gradient. Specifically, because competitive effects on species *A* were more environmentally sensitive than those on species *B*, fitness ratios increased along the gradient and caused competitive exclusion. This illustrates a microbe‐independent avenue for altered competitive outcomes along an environmental gradient.

The final set of theoretical simulations incorporated PSF with environmental context‐dependency (Figure 2C). As the environmental condition increases, the benefit received from the mutualist decreases, but unequally for these species. Ultimately, this results in priority effects when the environmental variable is low (with both symbionts behaving like mutualists), coexistence when the environmental variable is high (with both symbionts behaving like pathogens) and exclusion by species *A* at intermediate values for the environmental variable.

### Sensitivity analysis

#### Methods

To identify which types of species interactions may influence plant coexistence dynamics the most, we performed a local sensitivity analysis on a PSF model containing two competing plant species and one shared microbial taxon (SeAn model). The output of this analysis identifies whether the environmental‐dependence of particular species interactions might be more or less consequential for niche and fitness differences and plant coexistence. To do this, we sequentially varied each parameter, holding all other parameters constant, and computed the niche differences and fitness ratios across the parameter range. The baseline parameterization for this analysis was $c_{\mathrm{ii}}=-0.06$, $c_{\mathrm{ij}}=-0.055$, $c_{\mathrm{jj}}=-0.075$, $c_{\mathrm{ji}}=-0.05$, $\sigma_{\mathrm{iX}}=-0.002$, $\sigma_{\mathrm{jX}}=-0.00225$, $\phi_{\mathrm{Xi}}=10.5$, $\phi_{\mathrm{Xj}}=10$. The minimum and maximum parameter values used to constrain the sensitivity analysis were as follows: competition coefficients, $c_{\mathrm{ij}}=\left[-0.1,0\right]$; cultivation rates, $\phi_{\mathrm{Xi}}=\left[0,20\right]$; and microbial effects on plants, $\sigma_{\mathrm{iX}}=\left[-0.01,0.01\right]$. These distributions represent reasonable parameter values, but their magnitudes likely depend on the life history strategy of the microbial taxon. Specifically, our parameterization represents microbial taxa that are cultivated rapidly but that have small per‐capita (per‐cell) effects on plants. Alternatively, there may be rare but impactful microbial taxa, or one could parameterize this model in terms of hundreds or thousands of cells, in which case reasonable ϕ values would be relatively small and σ values would be much larger. Because the relative importance of ϕ and σ is dependent on these parameterization decisions (for which there is little empirical research to guide), we also present the sensitivity of niche and fitness differences to the product of $\phi_{\mathrm{ix}}$ and $\sigma_{\mathrm{xj}}$ which represents the indirect effect of a plant on its competitor via the cultivation of the microbial taxon (i.e., the plant–microbe feedback). We notate this plant–microbe feedback term as $m_{\mathrm{ij}}$ and set the minimum and maximum values for this parameter as −0.2 and 0.2 respectively (i.e., the minimum and maximum possible values based on the ranges of ϕ and σ defined above). Appendix S3 contains information about these sensitivities, including a sensitivity analysis of the fitness inequalities, which are the maximum value of the fitness ratios for the two species, a comparison of these results to those of a partial derivative‐based local sensitivity analysis and a simulation‐based global sensitivity analysis, and a comparison of the sensitivity results across a gradient of microbial taxonomic richness.

#### Results

**FIGURE 3:**
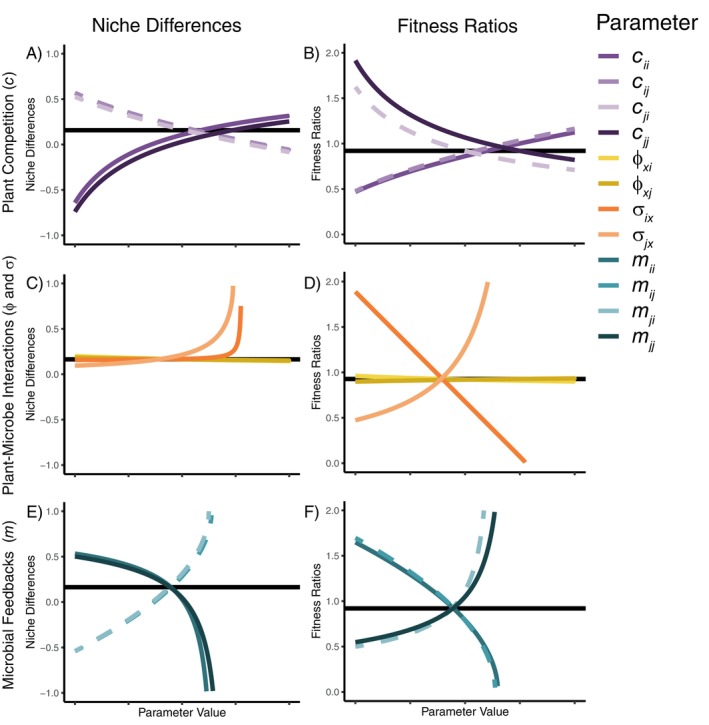
Visual sensitivity analysis of the taxon‐specific plant–soil feedback Lotka‐Volterra model. Lines represent the changes in niche differences (A, C, and E) and fitness ratios (B, D, and F) as the focal parameter varies from its minimum to maximum value while all other parameters are held constant at their baseline values. Dashed lines represent interspecific plant interactions. Panels A and B show sensitivity to microbe‐independent competition, panels C and D show sensitivity to plant–microbe interactions, and panels E and F show sensitivity to microbial feedbacks (i.e., the products of the plant–microbe interactions). Horizontal black lines show the value for the niche differences or the fitness ratio at the baseline parameterization, so the point at which each curve overlaps with the horizontal line is the position of the baseline parameter on the standardized axis. Some curves appear truncated as the value of the parameter gets closer to pushing the overall interaction terms (α) into facilitation, for which our definitions of niche differences and fitness ratios are undefined.

The component species interactions in our theoretical PSF model vary in their relative contribution to niche differences and fitness ratios (Figure 3). The niche differences monotonically increased (in the case of all plant–microbial interaction terms and the intraspecific competition coefficients) or decreased (in the case of interspecific competition coefficients). The general steepness of each of these curves (sensitivity) depended on the focal parameter identity and value (Figure 3A,C,E). Shifts in plant–plant interactions resulted in steep shifts in niche differences, whereas plant–microbe interactions had a smaller influence on niche difference (aside from a boundary behavior as the σ values push overall interaction coefficients closer to zero). Finally, increasing the value of the pathogenic microbial feedbacks (m) (thus decreasing the strength of the overall feedback as they become less negative and approach zero) impacted the niche differences more so than direct plant–microbe interactions (σ and ϕ) but less so than microbe‐independent competition (c). Interspecific pathogenic feedbacks increased niche differences as they weakened in strength whereas intraspecific pathogenic feedbacks decreased niche differences as they weakened in strength.

Distinct from the niche differences, fitness ratios monotonically increased with those species interactions that decreased the fitness of the competitively inferior species and decreased with those that decreased the fitness of the competitively superior species (Figure 3B,D,F). Further, the fitness ratios were much more sensitive to microbial effects on plants (σ) and least sensitive to plant cultivation rates (ϕ) throughout much of the range of the focal parameter. The fitness ratios had intermediate sensitivity to the plant–plant interaction coefficients and the microbial feedback terms. Relationships between parameter values and the fitness inequalities are explored in Appendix S3 (Figure S1). While most of these results were consistent across different types of sensitivity analyses, the importance of direct plant competition and the importance of microbial feedbacks, as measured by the partial derivative‐based local sensitivity analysis, did not differ (Appendix S3: Figure S3).

### Application to a species invasion

#### Methods

To explore how these theoretical findings that environmental conditions can mediate the effects of PSFs on plant coexistence may help inform empirical examples, we altered simulations based on recent work on an invasive plant, *A. petiolata*, as there is a general hypothesis that context‐dependencies in PSFs may help facilitate plant invasions and range‐expanding native species (van der Putten et al., 2016). *A. petiolata* was introduced to North America from Europe, causing decreased local native plant diversity and altered community structure in northeastern hardwood forests (Rodgers et al., 2008). This invasion is a useful case study because of the well documented shifts in microbial community composition in response to *A. petiolata* invasions (Anthony et al., 2019; Duchesneau et al., 2021). Additionally, extensive research has been conducted on the potential for allelopathic chemicals produced by *A. petiolata* to inhibit mutualisms between competing plants and soil microbes (Cipollini, 2016; Wolfe et al., 2008). Recent work has called for increased attention to the context dependencies of species interactions involved in *A. petiolata* invasions (Rodgers et al., 2022). For example, Blossey et al. (2021) found that *A. petiolata* growth rates decrease over time after initial invasion and hypothesized that this decrease might be due to a negative PSF. Further, the rate of decrease for *A. petiolata* growth rates varied by geographic location, suggesting that environmental context might alter the dynamics of the PSF.

We applied our modeling approach to explore how these geographically distinct PSFs following invasion by *A. petiolata* may arise (Blossey et al., 2021). We started with a baseline scenario in which, under sterile conditions, one species is competitively dominant over the other, and then introduced the effects of environment‐dependent PSFs. Microbes were introduced with increasing functional complexity (different microbial guilds) including: (1) a species‐specific pathogen affecting the competitively dominant species, *A. petiolata* (InSi I), (2) the same species‐specific pathogen and an additional species‐specific mutualist impacting the native, competitively inferior species (InSi II), and (3) both the species‐specific pathogen and mutualist as well as a generalist decomposer (InSi III). Finally, in each of these simulations, we calculated coexistence outcomes along a precipitation gradient, with the environmentally dependent cultivation parameter ($\phi_{\mathrm{XI}}$). Because we used linear scaling functions, we set all negative values of ϕ to zero. We provide an overview of scenarios here; full details and parameterizations can be found in the Appendix S4. Note that because the cultivation rate cannot fall below zero, we use a piece wise function that scales linearly when the output of the scaling function is ≥0 and remains at 0 otherwise.

We first considered a simplified set of “key players” in the system, where *A. petiolata* (*A*) competed with a native competitor (*B*) and cultivated a species‐specific pathogen (*X*). Thus, *A. petiolata*'s population growth rate was calculated as

(9) $$\frac{{\mathrm{dN}}_{A}}{\mathrm{dt}}=r_{A}N_{A}\left(1+c_{\mathrm{AA}}N_{A}+c_{\mathrm{AB}}N_{B}+\sigma_{\mathrm{AX}}S_{X}\right)$$

and the growth rate of the native competitor was calculated as

(10) $$\frac{{\mathrm{dN}}_{B}}{\mathrm{dt}}=r_{B}N_{B}\left(1+c_{\mathrm{BB}}N_{B}+c_{\mathrm{BA}}N_{A}\right)$$

such that the pathogen affected the growth rate of *A. petiolata* but not that of the native competitor.

As a baseline, we parameterized the models such that at some environmental average, and in sterile conditions, *A. petiolata* outcompetes its native competitor. Thus, the $c_{\mathrm{ij}}$ terms were set such that $|c_{\mathrm{AA}}|<|c_{\mathrm{BA}}|$ and $|c_{\mathrm{BB}}|>|c_{\mathrm{AB}}|$ (absolute values because we define competitive interactions as negative c values). The parameters for $\sigma_{\mathrm{AX}}$ and $\phi_{\mathrm{XA}}$ were selected such that at average environmental conditions, the pathogenic effect on *A. petiolata* enabled species coexistence. Finally, we used a linear scaling function to define how the cultivation of the pathogen by *A. petiolata* increases with increased soil moisture.

We next added two additional taxa, increasing model complexity and realism. First, we added a species‐specific mutualist for the native competitor (InSi II). Based on the response of mycorrhizal fungi to drought found by Lozano et al. (2021), we defined a scaling function such that the cultivation of the mutualist by the native competitor decreases as soil moisture increases. Lastly, we added a generalist decomposer that benefited both species (via, e.g., nutrient mineralization; InSi III) and its cultivation by both species increased as soil moisture increases.

For all simulations, we calculated $\alpha_{\mathrm{ij}}$ terms, fitness ratios, and niche differences in sterile and live conditions and under a range of soil moisture values. From these values, we examined how the context‐dependency of plant–microbe interactions were predicted to alter competitive outcomes between *A. petiolata* and the native competitor.

#### Results

**FIGURE 4:**
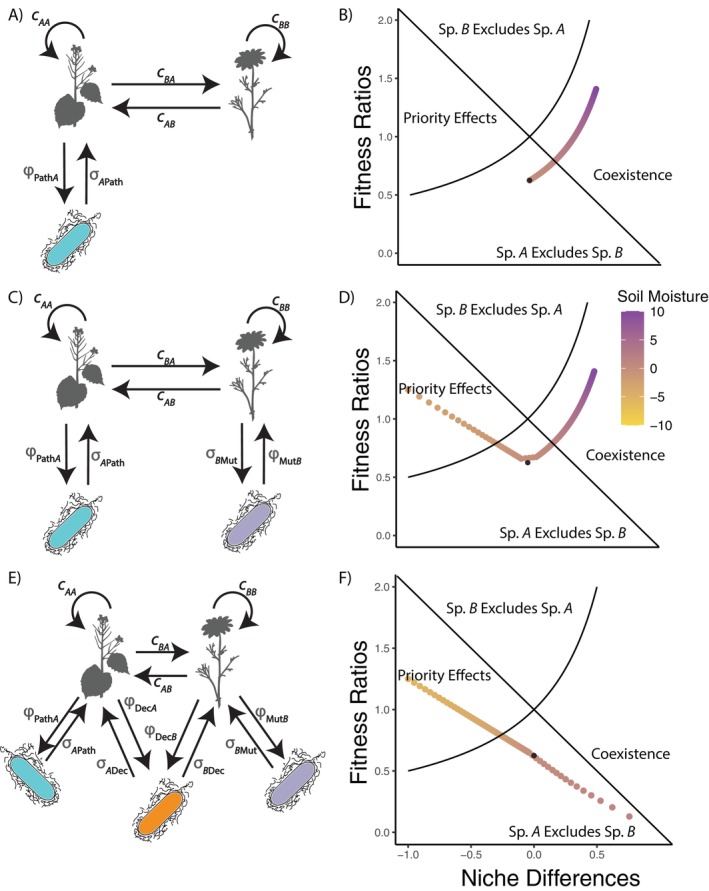
Model structure (left column) of the three *Alliaria petiolata* competition models alongside their simulated competition outcomes across a soil moisture gradient (right column). (A) A species‐specific pathogen is both cultivated by ($\phi_{\mathrm{XA}}$) and negatively impacts ($\sigma_{\mathrm{AX}}$) *A. petiolata*, stabilizing competition between *A. petiolata* and the native competitor. (B) Cultivation of the pathogen causes the two species to coexist at high soil moisture, whereas at low soil moisture *A. petiolata* outcompetes the native. (C) Adding to the previous model, a species‐specific mutualist both cultivates ($\phi_{\mathrm{YB}}$) and positively affects ($\sigma_{\mathrm{BY}}$) the native competitor. (D) Depending on soil moisture levels, priority effects (low soil moisture), competitive exclusion, or coexistence (high soil moisture) is predicted. (E) The last model builds upon previous models by including a generalist decomposer that benefits both *A. petiolata* ($\sigma_{\mathrm{AZ}}$) and the native competitor ($\sigma_{\mathrm{BZ}}$) and is cultivated by both *A. petiolata* ($\phi_{\mathrm{ZA}}$) and the native competitor ($\phi_{\mathrm{ZB}}$). (F) Cultivation of the decomposer by both *A. petiolata* and the native competitor yields priority effects or competitive exclusion of the native, depending on soil moisture. Black dots on the right column panels represent the niche differences and fitness ratios in the absence of microbes. This dot deviates from the curve when the intercept of the scaling functions of multiple microbial terms are unequal (such that simulated microbial systems never become equivalent to sterile conditions). Organism silhouettes retrieved from PhyloPic and modified. Species *A* uses *A. petiolata* by Michelle R. Jackson, and the microbe is *Sonnebornia yantaiensis* by Matt Crook; both are used under an Attribution‐ShareAlike 3.0 Unported license (https://creativecommons.org/licenses/by‐sa/3.0/). Species *B* is *Chamaemelum fuscatum* by David García‐Callejas under a CC0 1.0 Universal Public Domain Dedication license.

In the InSi I model, we found that the ability of pathogens to shift competitive outcomes between the native species and competitively superior invader from exclusion to coexistence depended on soil moisture (Figure 4B). At low soil moisture levels, the pathogen was not cultivated quickly enough to confer a stabilizing effect on the invasive species. However, as soil moisture increased, the cultivation of the pathogen occurred more rapidly, resulting in higher overall intraspecific competitive effects for the invasive species. At high soil moisture levels, stabilization by the pathogen was sufficient to result in coexistence.

When adding a species‐specific mutualist that interacts with the native species in the InSi II model, increasing soil moisture not only increased the cultivation of the species‐specific pathogen, and thus the intraspecific competitive effect on the invasive species, but it also decreased the cultivation of the mutualists, increasing the intraspecific competitive effect on the native species (Figure 4D). Note that the cultivation rates are constrained to be greater than or equal to zero, creating the piecewise structure to Figure 4D when one or both cultivation rates reach and remain at zero. Thus, at high soil moisture levels, the dynamic still results in coexistence, as in the simulations with no mutualist, but the decreased cultivation of the mutualist equalizes the interaction by keeping fitness ratios closer to zero. Another new outcome from these simulations is that at low soil moisture levels, the decreased cultivation of the pathogen and the increased cultivation of the mutualist result in priority effects. Therefore, the combined alleviation of the invasive species' negative soil feedback and the increase in the native species' positive soil feedback can actually destabilize coexistence, where competitive exclusion now depends on species arrival order.

Finally, we examined how the addition of a generalist microbial decomposer in the InSi III model, cultivated by and providing nutrient access to both plant species, may impact coexistence (Figure 4E). Positively scaling the cultivation of the decomposer by both species with soil moisture resulted in no stable coexistence predicted along the soil moisture gradient (Figure 4F). At low soil moisture levels, the decreased cultivation of the decomposer resulted in similar priority effects from the previous set of simulations. Increasing the soil moisture increased the niche differences and decreased fitness ratios, eventually resulting in the exclusion of the native species by the invasive species.

## DISCUSSION

The strength and effect of species interactions, including PSFs, can depend strongly on underlying environmental conditions, with the magnitude and even direction (e.g., competitive vs. facilitative) of interactions being dependent on the environment (Hahn et al., 2018; Nuske et al., 2021). In order to incorporate the widely observed environmental context dependencies into models of PSFs and determine the effects on species coexistence, we have explicitly modeled distinct microbial taxa instead of whole communities and allowed key PSF parameters to vary with the environment. Simulations support the mounting empirical evidence that context‐dependent, inter‐guild species interactions may influence the outcome of competition (Karakoç et al., 2020; Thurman et al., 2019). Our sensitivity analysis suggests that while niche differences are generally more sensitive to shifts in direct plant competition than shifts in plant–microbe interactions, the fitness ratios are generally more sensitive to shifts in plant–microbe interactions. Further, the invasive species simulations suggest that even simple context‐dependent interactions can flip coexistence outcomes along an environmental gradient, especially when including multiple microbial guilds, as commonly occur in nature. These findings indicate that ignoring these context‐dependent species interactions, as many empirical studies do for logistical reasons, likely leads to erroneous conclusions about competitive dynamics and suggest caution in extending results from one environmental context to other scenarios. Overall, these results suggest several lines of future research needed to adequately incorporate microbial effects into plant coexistence studies and highlight the importance of ecological context dependencies beyond single‐trophic levels.

The *A. petiolata*‐inspired simulations highlight key issues in the biogeography of invasive species. First, these simulations suggest that site‐specific environmental conditions (e.g., soil moisture) coupled with PSF could be responsible for the region‐specific rates of decline in *A. petiolata* growth rates reported by Blossey et al. (2021). Identification of the key microbial taxa causing the negative PSF and research on their responses to spatial heterogeneity is likely to provide further insight into the invasion dynamics of *A. petiolata* across North America. Second, these results implicate context‐dependent species interactions as one reason for inconsistencies across studies of invasive species. Although most hypotheses for invasion success explicitly acknowledge spatial differences (either biotic or abiotic) between invaders' home range and invaded range (Enders et al., 2020), most of these hypotheses have received deeply conflicting support (Gallien & Carboni, 2017). Further, few hypotheses explicitly acknowledge spatial differences within the invaded range. This may, in part, be due to spatial heterogeneity at multiple scales influencing invasion success through context‐dependent biological interactions. For example, our findings are consistent with research that has shown how the enemy release of invasive species can be driven by context‐dependent interactions with soil microbes (McGinn et al., 2018; Nuske et al., 2021). Recognition of context‐dependent interactions between invasive species and soil microbes is likely to help resolve apparent contradictions within traditional invasion biology hypotheses.

The sensitivity analyses indicated that shifts in the component species interactions of PSFs do not equally affect competitive dynamics between plant species. Most notably, we found that while niche differences were generally more sensitive to direct plant competition than plant–microbe interactions, microbial effects on plants affected fitness ratios more so than direct plant competition, demonstrating the importance of microbe‐induced fitness ratios (Kandlikar et al., 2021; Yan et al., 2022). Conversely, the local sensitivity of niche differences and fitness ratios, while certainly dependent on baseline parameter values, were the same with respect to microbe‐independent competition and microbial feedbacks, highlighting the dependency of sensitivity results to the exact parameterization of the PSF model. Nevertheless, the direct plant–microbe sensitivity results are consistent with recent assessments of multitrophic communities that identified trophic‐level‐specific relative importances of niche versus fitness differences (Song & Spaak, 2023, 2024). The mathematic mechanism for our results involves the additive function within the numerator of the partial derivative of the niche differences with respect to σ (Appendix S3: Equations 7 and 8), which is absent from that of the fitness ratio (Appendix S2: Equations 15 16). Ecologically, these microbial terms largely determine how species might differ in the degree to which they can capitalize on or are negatively affected by the microbial community that both species are experiencing. Thus, shifts in these microbial‐plant species interactions are more so related to the difference in the baseline fitness of species rather than potential differentiation of niche space, as might occur under specialized plant–microbe interactions.

Importantly, network characteristics of the coupled plant–microbe community may determine how context‐dependent PSFs mediate plant coexistence. Microbial taxonomic richness, species specificity of plant–microbe interactions, and the exact sign and strength of species interactions are all likely to impact specific mechanisms of plant coexistence, and while it is beyond the scope of this paper to test each of these, we do show how variation in some of these network characteristics influences coexistence in the simulations. Further, we tested whether microbial taxonomic richness impacts the inference we have made about distinctive roles of plant–plant interactions and plant–microbe interactions in determining niche differences and fitness ratios. We found little evidence that taxonomic richness per se influences these key findings. However, other unexplored aspects of network structure may still complicate these findings. Notably, the importance of σ as opposed to ϕ is a result of the baseline parameterization which reflects our assumption that microbes have high equilibrium densities and low per‐capita impacts on plants (i.e., any one microbe has relatively weak impacts on plants). While the particular plant–microbe interaction (ϕ or σ) which impacts the fitness ratio depends on this assumption, the general finding that one of these terms influences fitness differences more so than direct plant–plant interactions do remains constant across a variety of parameterizations. It is also worth noting that all sensitivity analyses require a priori assumptions about parameters, many of which are untested in plant–microbe communities. Research on general patterns of coupled plant–microbe networks may guide similar analyses in the future.

In addition to spatial context‐dependencies, plant competition and invasion dynamics are likely to exhibit temporal context‐dependencies, given annual climatic variation and now rapid climate change. Directional shifts in annual climate variables such as temperature, drought, and atmospheric CO_2_ levels, are predicted to alter the dynamics of soil microbial communities (Pugnaire et al., 2019). Greenhouse studies have found support for drought‐induced legacy effects on PSF's but have found no support for temperature‐dependent PSFs Gundale and Kardol (2021) and Kaisermann et al. (2017). However, Van Grunsven et al. (2010) suggest that future research on the effect of climate change on PSFs should acknowledge many interacting abiotic and biotic factors. Although these models highlight how directional changes in mean environmental conditions (e.g., via climate change) may impact coexistence outcomes, interannual variation may also be important (Rudgers et al., 2020). Thus, future modeling efforts should extend our approach to modeling context‐dependent PSFs by incorporating the potential for fluctuation‐dependent stabilization.

Predicted shifts in temporal autocorrelation of environmental variables (e.g., temperature) are likely to influence species interactions (Di Cecco & Gouhier, 2018; Gudmundson et al., 2015). These fluctuation‐dependent mechanisms of species coexistence have been a cornerstone of MCT (Chesson, 2000). However, our work shows that even if plant–plant interactions are constant across an environmental gradient, context‐dependent PSFs may also impact coexistence outcomes. Further, both are likely simultaneously operating in many communities. Because the derivation of niche and fitness ratios we have used is restricted to fluctuation‐independent mechanisms of coexistence (Chesson, 2013), other approaches such as Ellner et al.'s (2019) decomposition of invader growth rates might be appropriate for extending this framework to incorporate variability in drivers and fluctuation‐dependent mechanisms of coexistence. This method has been used to show how other inter‐guild interactions mediate species coexistence and may offer novel insight into interactions between environmental variability and PSFs (Shoemaker et al., 2020).

While many biological processes may respond in nonlinear ways to environmental variation, we found that linear approximations may be sufficient for understanding how environmentally mediated interactions impact competitive interactions, depending on the scale at which systems vary along the gradient (Appendix S1). However, the linear effects of the environment on interaction strengths in our models resulted in striking nonlinear consequences for fitness ratios and niche differences (Figures 2 and 4), emphasizing the complex interdependence between these two axes of coexistence (Song et al., 2019). Other work investigating coexistence has found similar nonlinear trends in these axes of coexistence for both single‐trophic (Bowler et al., 2022) and multitrophic systems (Terry et al., 2021). This may suggest that predictions based on two discrete experimental conditions (ambient vs. warmed, low vs. high precipitation, etc.) provide inadequate information to extrapolate outside of these conditions or even to intermediate conditions that fall between discrete measurements. Further integration of this theoretical framework and empirical studies could help decompose the direct and indirect PSF effects on both niche differences and fitness ratios.

Though widely recognized as a nearly universal phenomenon, to date context‐dependency has not been studied with a coherent and generalizable methodology capable of quantifying the role of particular environmental dependencies on PSF mediated community dynamics. Ke and Wan (2022) previously proposed a method of parameterizing their models that may be extended to include these context‐dependent models of PSF across environmental conditions. Briefly, they suggested a greenhouse study in which microbe‐independent competition coefficients ($c_{\mathrm{ij}}$) can be disaggregated from microbial contributions ($\sigma_{\mathrm{iX}}$ and $\phi_{\mathrm{Xi}}$) to the overall competition coefficients ($\alpha_{\mathrm{ij}}$) by growing plants alone and along a competition gradient in either sterile or competitor‐conditioned soil. To assess the role of a given environmental context‐dependency, this experiment could be repeated along an environmental gradient, where these parameters could be modeled as a function of the environmental variable. One obstacle to empirically estimating the role of context‐dependent species interactions using this approach is identifiability constraint of σ and ϕ. Molecular techniques to estimate the cultivation rate ϕ may be implemented to either quantify that absolute abundance (qPCR), microbial load (hamPCR), or shifts in community composition (amplicon sequencing) along plant density gradients. The link between the presented models and potential experimental approaches is one benefit of the use of phenomenological interaction coefficients. However, to tackle the more mechanistic questions related to context‐dependent PSF‐mediated coexistence, molecular methods such as transcriptomics might be used to disentangle whether compositional shifts in microbial communities or functional shifts in microbial taxa are responsible for these context‐dependent effects (Schenk et al., 2012; Xia et al., 2021; Yergeau et al., 2014). More generally, however, we suggest that PSF studies would benefit from incorporating modeling approaches and experimental designs that allow for context‐dependent species interactions.

Recent work has shown how coexistence theory and PSF concepts can be productively integrated to better understand species coexistence (Kandlikar, 2024; Kandlikar et al., 2019, 2021; Ke & Wan, 2020; Yan et al., 2022). The dual role of niche differences and fitness ratios in mediating the outcome of species interactions offers a more nuanced and mechanistic understanding of both positive and negative PSFs that should inform empirical studies. PSFs and their context‐dependencies may be responsible for spatiotemporal trends that previous researchers have noted in niche differences and fitness ratios (Hallett et al., 2019; Sears & Chesson, 2007), and may be a key mechanism mediating coexistence across communities. Our results paired with the ample empirical evidence of abiotic context‐dependent plant–microbe interactions (Bennett & Klironomos, 2019; Gundale & Kardol, 2021) highlight the potential explanatory power of these particular mechanisms, which have the potential to explain cases in which theoretical models of coexistence fail to predict empirical results (Kraft et al., 2015; Wainwright et al., 2019).

## AUTHOR CONTRIBUTIONS

Jeremy A. Collings, Lauren G. Shoemaker, and Jeffrey M. Diez were all involved in the development of the modeling framework. Jeremy A. Collings conducted all simulations, produced all figures, and wrote the first draft of the manuscript. Lauren G. Shoemaker and Jeffrey M. Diez provided feedback for subsequent revisions.

## CONFLICT OF INTEREST STATEMENT

The authors declare no conflicts of interest.

## Supporting information


Appendix S1.

Appendix S2.

Appendix S3.

Appendix S4.

## Data Availability

Code (Collings, 2025) is available in Zenodo at https://doi.org/10.5281/zenodo.15642842.
