# Supplementary material for: Environmental context alters plant–soil feedback effects on plant coexistence
Source: Ecology. 2025 Aug 6;106(8):e70170. doi: 10.1002/ecy.70170 (PMC12327179; doi:10.1002/ecy.70170)
Supplement: Supplementary file 1 — Appendix S1. [file ECY-106-e70170-s002.pdf]

Appendix S1: Non-linear Responses to Environmental Variables

Environmental context alters plant-soil feedback effects on plant co-existence

Jeremy A. Collings, Lauren G. Shoemaker & Jeffrey M. Diez

in *Ecology*

In the core simulations in this study, we assumed linear relationship between the environment and species interactions. However, it's possible that species interactions have saturating or parabolic functional responses to relevant environmental variables. If some environmental variable monotonically affects the interaction strength, it is possible that some upper or lower limit imposes a saturating functional relationship. Alternatively, optimum environmental conditions may impose a parabolic function relationship. Here, we explore these relationships and assess the consequences of nonlinearity in the models described in the main text.

1 Methods

To assess how nonlinear responses of plant-microbial interactions to the environment may affect plant coexistence, we reran the baseline empirical invasion scenario with three forms of the scaling functions (linear, sigmoidal, and parabolic) for both the cultivation rate of the pathogen ϕ_{XA} and the microbial effect on the pathogen on the invasive plant σ_{AX}. To further explore non-monotonic scaling functions, we also examined scaling functions of σ_{AX} that span both sides of the parabola in three different scenarios.

1.1 Comparison of Nonlinear Forms

For each of these simulations, two plant species are competing with one another, and the competitively dominant plant species cultivates a species-specific pathogen. The microbe-independent competition coefficients are set as $c_{AA} = -0.5$, $c_{BA} = -0.8$, $c_{BB} = -0.8$, and $c_{AB} = -0.5$. We then proceeded to sequentially replace static parameter values for ϕ_{XA} and σ_{AX} with linear, sigmoidal, and parabolic functions of an environmental gradient, while keeping the value of the other plant-microbe interactions static at a baseline value. When σ_{AX} was scaled by the environmental gradient, ϕ_{XA} was held constant at 0.2, When ϕ_{XA} was scaled, σ_{AX} was held constant at -0.2. Sigmoidal and parabolic scaling functions were

designed to have similar minimum and maximum values along the range of the moisture gradient to that of the linear scaling functions (Table S1).

Table S1: Equations for Comparisons of Nonlinear Scaling Functions

Function	Parameter	Equation
Linear	ϕ_{XA}	$\phi_{XA} = .2 + v$
	σ_{AX}	$\sigma_{AX} = -.2 - v$
Sigmoidal	ϕ_{XA}	$\phi_{XA} = \frac{2}{1+e^{-2v}}$
	σ_{AX}	$\sigma_{AX} = \frac{4}{1+e^{2v}} - 2.2$
Parabolic	ϕ_{XA}	$\phi_{XA} = -.5(v-2)^2 + 2.2$
	σ_{AX}	$\sigma_{AX} = .25(v-2)^2 - 2.2$

1.2 Further Exploration of Parabolic Scaling

For the additional parabolic scaling functions of σ_{AX}, we began with a scaling function of the one species-specific pathogen's effect on the competitively dominant plant species with the vertex (i.e. the point along the rainfall gradient where the pathogencity is the lowest) at zero:

$$\sigma_{XA} = .25v^2 - 2.2 \tag{1}$$

We then added an additional species-specific pathogen on the competitively inferior species that is scaled identically to the first pathogen. Finally, we reran these simulations after adjusting the new pathogen's scaling function to offset its minimum pathogencity from the original pathogen:

$$\sigma_{YB} = .25(v-1)^2 - 2.2 \tag{2}$$

For all simulations, the cultivation rate was kept positive by dropping negative outputs of the ϕ_{XA} and ϕ_{YB} scaling functions from subsequent calculations. After scaling, we calculated the overall competition coefficients as $\alpha_{ij} = c_{ij} + \sigma_{iX}\phi_{Xj}$. Across all scenarios, we calculated niche and fitness differences along the environmental gradient to quantify the downstream effects of these different forms of environmental context dependency on coexistence dynamics.

2 Results and Discussion

Sigmoidal and half-parabolic scaling functions did not dramatically differ in their downstream effects on niche and fitness ratios from those of the linear scaling functions (Figure S1). Importantly, when these scaling functions are monotonic and span a similar range of parameter values, the overall trajectory through niche and fitness ratios space is similar. However, non-linearities do shift the rate of change in these values such that the sensitivity of the context-dependent parameter (and thus the competitive dynamics) is dependent on the position along the environmental gradient.

In our exploration of full parabolic functions, we do find novel shifts in the trajectory which were not observed in other sets of our simulations (Figure S2). When one pathogen's infleunce on a host species is parabolically dependent on rainfall, the plant competitive dynamics move parabolically through niche/fitness space such that they move into coexistence space at the vertex of the scaling function and then retrace the initial trajectory in reverse (Figure S2a). This behavior is similarly seen when two species have rainfall-dependent sensitivities to pathogens, but only when these scaling functions share a vertex (Figure S2b). When these scaling functions do not share a vertex, the trajectory is not retraced, and at each point along the environmental gradient, the species pair exists at a unique point in niche/fitness space (Figure S2c).

This brief case study in these nonlinear context-dependencies highlights an important consideration for the modeling of context-dependent species interactions: the form of these models is determined, in part, by the scale along the environmental gradient. This points to two seemingly simple but nonetheless useful insights. First, across a small enough range along an environmental gradient, linear approximations can make nearly identical predictions about the behavior of the community. Thus, effort should be focused on assessing parameter values across the environmental gradient, but determining the exact functional form may be a lower priority. Second, general information about the form of the scaling function at larger scales can inform our expectations about shifts in the velocity and trajectory of system dynamics. For example, parameters with saturation scaling functions will likely have a smaller rate of change with respect to the environmental variable at high extremes of the gradient. Alternatively, parameters with a local optimum are likely to shift in their velocity as well as the trajectory of the competitive dynamics through niche and fitness difference space as the environmental variable changes.

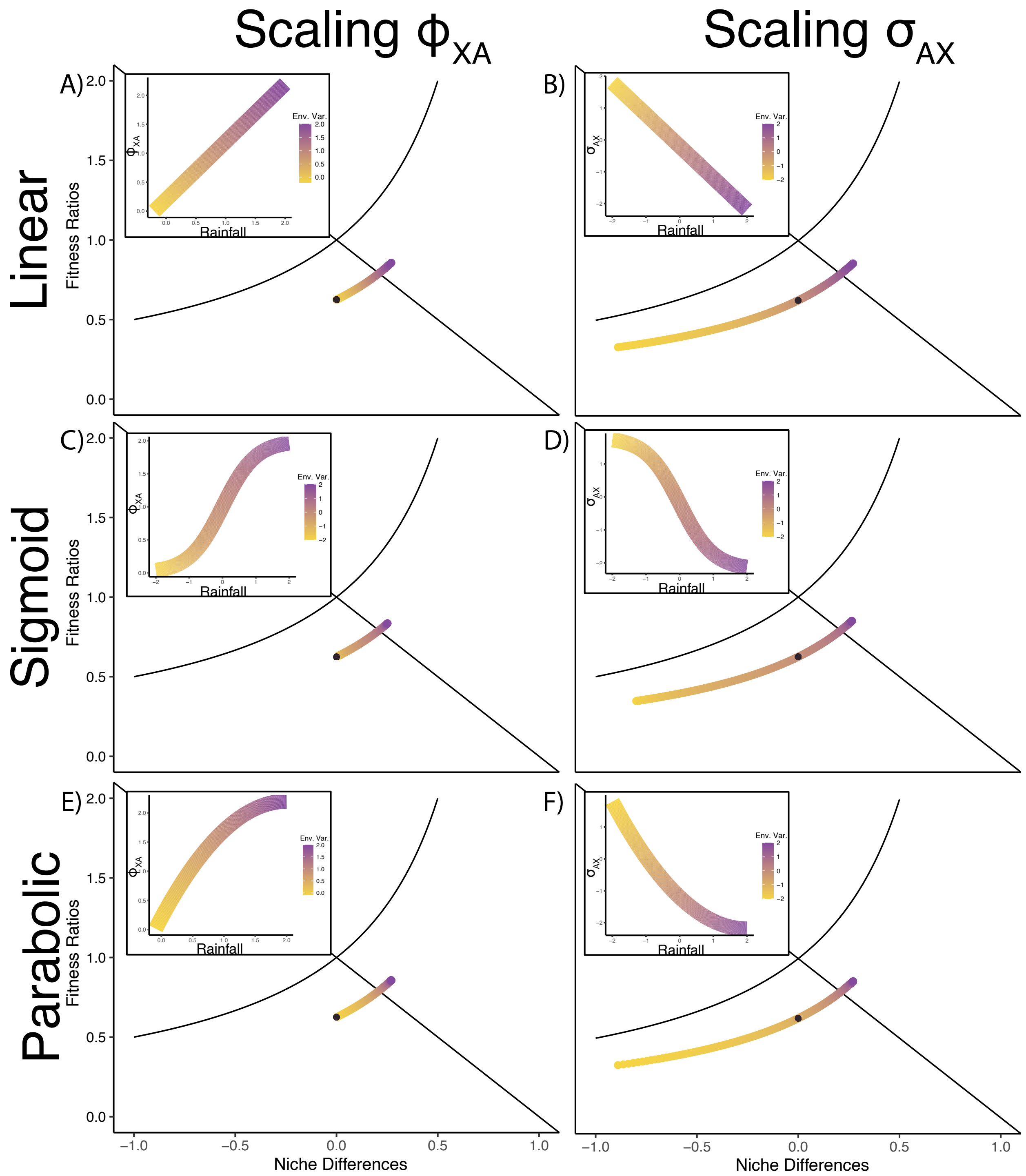

Figure S1: Results from linear (A & B), sigmoidal (C & D), and parabolic (E & F) scaling functions for ϕ_{XA} (first column; A, C, and E) and σ_{AX} (second column; B, D, and F). Graphs of the scaling functions are inlaid into each plot in niche difference and fitness ratio space.

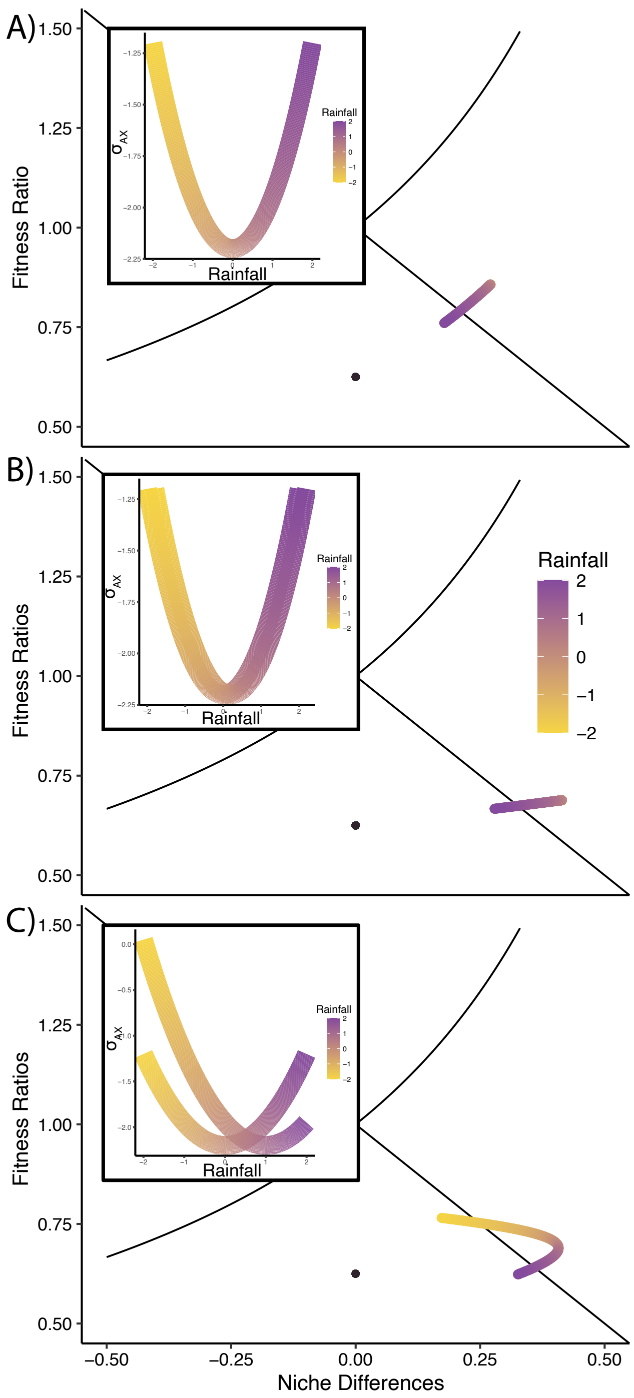

Figure S2: Effects of full parabola scaling functions for σ_{AX} and σ_{BY} on niche differences and fitness ratios. A) A single species-specific pathogen with a minimum pathogenicity on the competitively dominant species at 0 along the rainfall gradient. B) Two species-specific pathogens, one for each plant species, with identical scaling functions with a minimum pathogencitiy at 0 along the rainfall gradient. C) Two species-specific pathogens with offset minimum pathogenicity values. Graphs of the scaling functions are inlaid into each plot in niche difference and fitness ratio space.
