# Supplementary material for: Environmental context alters plant–soil feedback effects on plant coexistence
Source: Ecology. 2025 Aug 6;106(8):e70170. doi: 10.1002/ecy.70170 (PMC12327179; doi:10.1002/ecy.70170)
Supplement: Supplementary file 2 — Appendix S2. [file ECY-106-e70170-s003.pdf]

Appendix S2: List of Model Structures in Main Text

Environmental context alters plant-soil feedback effects on plant coexistence

Jeremy A. Collings, Lauren G. Shoemaker & Jeffrey M. Diez

in *Ecology*

In this paper, we explore multiple structures of coupled plant-microbe communities. Ignoring the exact magnitude and sign of the species interactions, these structures vary in the taxonomic richness of the microbial community as well as the specificity of particular plant-microbe interactions. Actual plant-microbe community structures are incredibly diverse, due in part to the incredible diversity of microbes (Dundore-Arias *et al.*, 2023), and a large set of structures can be defined even with our greatly simplified communities with at most three microbial taxa. As a demonstrative example, we might constrain the set of network structures for our purposes to include only those with 1) two plant species which directly compete with one another as well as themselves, 2) at least one microbe which is both cultured by some plant (ie assuming all microbes require the presence of at least one plant) and affects the fitness of some plant (ie ignoring microbes which do not affect the fitness of either plant species). We can further reduce the set of all unique structures by ignoring the identity of the plants or the microbes. Just considering such directed, unweighted graphs produces 5, 25, and 85 unique structures for 1, 2, and 3 microbe species systems.

Our results demonstrate that the structure of these networks may impact plant community dynamics. Though not central to the goal of this paper, we do attempt to address this structural sensitivity by exploring multiple network structures in each of our simulations as well as in our sensitivity analysis. Here, we present a list of network structures used in the main text.

Diagram	Description	Name	Analysis
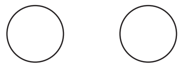	No plant-microbe interactions	ThSi I	Theoretical Simulations
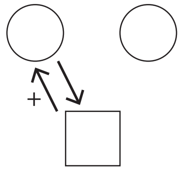	One specialist mutualist	ThSi II	Theoretical Simulations
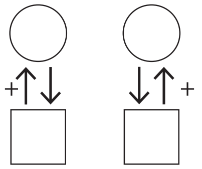	One specialist mutualist per species	ThSi III	Theoretical Simulations
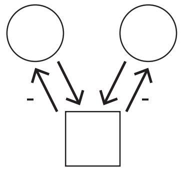	One generalist pathogen	SeAn	Sensitivity Analysis
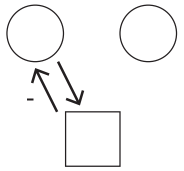	One specialist pathogen	InSi I	Invasion Simulations
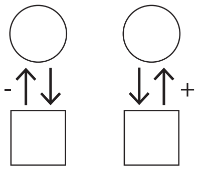	One specialist pathogen and one specialist mutualist	InSi II	Invasion Simulations

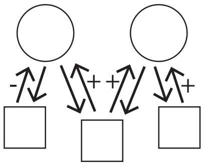	One specialist pathogen, one specialist mutualist, and one generalist mutualist/decomposer	InSi III	Invasion Simulations

References

Dundore-Arias, J., Michalska-Smith, M., Millican, M. & Kinkel, L. (2023). More than the sum of its parts: unlocking the power of network structure for understanding organization and function in microbiomes. *Annual Review of Phytopathology*, 61, 403–423.
