# Supplementary material for: Environmental context alters plant–soil feedback effects on plant coexistence
Source: Ecology. 2025 Aug 6;106(8):e70170. doi: 10.1002/ecy.70170 (PMC12327179; doi:10.1002/ecy.70170)
Supplement: Supplementary file 3 — Appendix S3. [file ECY-106-e70170-s001.pdf]

Appendix S3: Additional Sensitivity Analyses

Environmental context alters plant-soil feedback effects on plant co-existence

Jeremy A. Collings, Lauren G. Shoemaker & Jeffrey M. Diez

in *Ecology*

Here, we present the methods and results from an analytical local stability analysis as well as a partial-rank correlation coefficient (PRCC) based global sensitivity analysis. Our analytical local sensitivity analysis evaluates the sensitivity of niche differences and fitness ratios to these direct and indirect species interactions at a given point in parameter space. Conversely, our global sensitivity analysis evaluates the sensitivity of niche differences and fitness ratios to species interactions across some defined finite parameter space and includes the interactive impacts of parameters on the niche differences and fitness ratios. Our simulation-based local sensitivity analysis presented in the main text does not include the interactive effects of parameters, but does show how sensitivity to a given species interaction term changes as we move along it's axis in parameter space. Lastly, we use the analytical local sensitivity analysis to explore the potential for model structure (ie plant-microbe network topology) to influence the results of our sensitivity analysis. Specifically, we examined how the sensitivity of particular parameters shifts as additional microbial taxa are included in the model.

Based on our definition of overall competition coefficients (Eq. 6), we can define extended equations for the niche differences, fitness ratios, and fitness inequalities.

The niche differences can be defined as:

$$ND = 1 - \sqrt{\frac{(c_{ij} + \sigma_{ix}\phi_{xj})(c_{ji} + \sigma_{jx}\phi_{xi})}{(c_{ii} + \sigma_{ix}\phi_{xi})(c_{jj} + \sigma_{jx}\phi_{xj})}} \tag{1}$$

The fitness ratio can be defined as:

$$FR = \sqrt{\frac{(c_{ij} + \sigma_{ix}\phi_{xj})(c_{ii} + \sigma_{ix}\phi_{xi})}{(c_{ji} + \sigma_{jx}\phi_{xi})(c_{jj} + \sigma_{jx}\phi_{xj})}} \tag{2}$$

Finally, the fitness inequalities can be defined as the maximum value of the two fitness ratios, or the fitness ratio where species i is the competitively inferior species.

To better understand the role of c, σ and ϕ parameters in determining the value of the niche differences, fitness ratios, and fitness inequalities, we performed three types of sensitivity analyses: a local stability analysis (presented in the main text), an analytical sensitivity analysis, and a partial rank correlation coefficient (PRCC) based sensitivity analysis. Here, we provide the results of the local stability analysis for the fitness ratios as well as the methods and results for the analytical and PRCC-based sensitivity analyses. We also repeat this process to look instead at the differential roles of direct plant competition, c, and microbial feedbacks, m, which are defined as the product of ϕ and σ in our one species model. The maximum value function introduces additional mathematical and statistical complexity to calculating the sensitivity of the fitness inequalities, so we did not apply these additional methods to the sensitivity of the fitness inequalities, and instead analyzed the sensitivity of the fitness ratios and niche differences. The baseline parameterization for all of these analyses was the same as that used for the local sensitivity analysis: $c_{ii} = -0.06$, $c_{ij} = -0.055$, $c_{jj} = -0.075$, $c_{ji} = -0.05$, $\sigma_{ix} = 0.002$, $\sigma_{jx} = 0.00225$, $\phi_{xi} = 10.5$, $\phi_{xj} = 10$.

1 Local Sensitivity Analysis

The local sensitivity analysis for the niche differences and fitness ratios are displayed in Figure 3 in the main text. Here, we show the local sensitivity analysis for the fitness inequalities (Figure S1). The maximum value function within the calculation of the fitness inequalities creates relationships between parameter values and fitness inequalities that contain a single minimum value and that are asymmetric about this inflection point . The sensitivity of the fitness inequalities to each parameter can thus be assessed by inspecting the steepness on either side of the inflection point. In contrast to the niche differences, fitness inequalities were most sensitive to microbial effects on plants (σ) and least sensitive to plant cultivation rates (ϕ), with intermediate sensitivity to plant-plant interaction coefficients and microbial feedback terms.

2 Analytical Sensitivity Analysis

We conducted a derivative-based analytical sensitivity analysis by calculating the partial derivatives of the full niche differences and fitness ratio equations (Eq S1 & S2) and then computed the value of these partial derivatives at the baseline parameterization (Figure S2). Again, we repeat this process to calculate the sensitivity of niche differences and fitness ratios to microbial feedbacks (Figure S3). Here, we report the equations for the partial derivatives and present their values at the baseline. Notably, the partial derivatives for the microbial feedback terms are the same as those of their respective microbe-independent plant competition coefficients (e.g. the partial derivative of the niche differences with respect to c_{ij} is the same as that with respect to m_{ij}). Congruent with the local sensitivity analysis reported in the main text and above, we find that plant competition terms (c) and microbial feedback terms (m) similarly influence niche and fitness difference. Conversely, fitness ratios are more

sensitive to the microbial effects on plants (σ) than niche differences, and both metrics are much more sensitive to σ terms than the cultivation terms (ϕ) in this parameterization.

2.1 Partial Derivatives

2.1.1 ϕ & σ Parameterization

Niche Differences

$$\frac{\partial ND}{\partial c_{ij}} = -\frac{c_{\mathrm{ji}} + \sigma_{\mathrm{jx}}\phi_{\mathrm{xi}}}{2\left(c_{\mathrm{ii}} + \sigma_{\mathrm{ix}}\phi_{\mathrm{xi}}\right)\left(c_{\mathrm{jj}} + \sigma_{\mathrm{jx}}\phi_{\mathrm{xj}}\right)\sqrt{\frac{\left(c_{\mathrm{ij}}+\sigma_{\mathrm{ix}}\phi_{\mathrm{xj}}\right)\left(c_{\mathrm{ji}}+\sigma_{\mathrm{jx}}\phi_{\mathrm{xi}}\right)}{\left(c_{\mathrm{ii}}+\sigma_{\mathrm{ix}}\phi_{\mathrm{xi}}\right)\left(c_{\mathrm{jj}}+\sigma_{\mathrm{jx}}\phi_{\mathrm{xj}}\right)}}} \tag{3}$$

$$\frac{\partial ND}{\partial c_{ji}} = -\frac{c_{\mathrm{ij}} + \sigma_{\mathrm{ix}}\phi_{\mathrm{xj}}}{2\left(c_{\mathrm{ii}} + \sigma_{\mathrm{ix}}\phi_{\mathrm{xi}}\right)\left(c_{\mathrm{jj}} + \sigma_{\mathrm{jx}}\phi_{\mathrm{xj}}\right)\sqrt{\frac{\left(c_{\mathrm{ij}}+\sigma_{\mathrm{ix}}\phi_{\mathrm{xj}}\right)\left(c_{\mathrm{ji}}+\sigma_{\mathrm{jx}}\phi_{\mathrm{xi}}\right)}{\left(c_{\mathrm{ii}}+\sigma_{\mathrm{ix}}\phi_{\mathrm{xi}}\right)\left(c_{\mathrm{jj}}+\sigma_{\mathrm{jx}}\phi_{\mathrm{xj}}\right)}}} \tag{4}$$

$$\frac{\partial ND}{\partial c_{ii}} = \frac{\left(c_{\mathrm{ij}} + \sigma_{\mathrm{ix}}\phi_{\mathrm{xj}}\right)\left(c_{\mathrm{ji}} + \sigma_{\mathrm{jx}}\phi_{\mathrm{xi}}\right)}{2\left(c_{\mathrm{ii}} + \sigma_{\mathrm{ix}}\phi_{\mathrm{xi}}\right)^2\left(c_{\mathrm{jj}} + \sigma_{\mathrm{jx}}\phi_{\mathrm{xj}}\right)\sqrt{\frac{\left(c_{\mathrm{ij}}+\sigma_{\mathrm{ix}}\phi_{\mathrm{xj}}\right)\left(c_{\mathrm{ji}}+\sigma_{\mathrm{jx}}\phi_{\mathrm{xi}}\right)}{\left(c_{\mathrm{ii}}+\sigma_{\mathrm{ix}}\phi_{\mathrm{xi}}\right)\left(c_{\mathrm{jj}}+\sigma_{\mathrm{jx}}\phi_{\mathrm{xj}}\right)}}} \tag{5}$$

$$\frac{\partial ND}{\partial c_{jj}} = \frac{\left(c_{\mathrm{ij}} + \sigma_{\mathrm{ix}}\phi_{\mathrm{xj}}\right)\left(c_{\mathrm{ji}} + \sigma_{\mathrm{jx}}\phi_{\mathrm{xi}}\right)}{2\left(c_{\mathrm{ii}} + \sigma_{\mathrm{ix}}\phi_{\mathrm{xi}}\right)\left(c_{\mathrm{jj}} + \sigma_{\mathrm{jx}}\phi_{\mathrm{xj}}\right)^2\sqrt{\frac{\left(c_{\mathrm{ij}}+\sigma_{\mathrm{ix}}\phi_{\mathrm{xj}}\right)\left(c_{\mathrm{ji}}+\sigma_{\mathrm{jx}}\phi_{\mathrm{xi}}\right)}{\left(c_{\mathrm{ii}}+\sigma_{\mathrm{ix}}\phi_{\mathrm{xi}}\right)\left(c_{\mathrm{jj}}+\sigma_{\mathrm{jx}}\phi_{\mathrm{xj}}\right)}}} \tag{6}$$

$$\frac{\partial ND}{\partial \sigma_{ix}} = -\frac{\frac{\phi_{\mathrm{xj}}\left(c_{\mathrm{ji}}+\sigma_{\mathrm{jx}}\phi_{\mathrm{xi}}\right)}{\left(c_{\mathrm{ii}}+\sigma_{\mathrm{ix}}\phi_{\mathrm{xi}}\right)\left(c_{\mathrm{jj}}+\sigma_{\mathrm{jx}}\phi_{\mathrm{xj}}\right)} - \frac{\phi_{\mathrm{xi}}\left(c_{\mathrm{ij}}+\sigma_{\mathrm{ix}}\phi_{\mathrm{xj}}\right)\left(c_{\mathrm{ji}}+\sigma_{\mathrm{jx}}\phi_{\mathrm{xi}}\right)}{\left(c_{\mathrm{ii}}+\sigma_{\mathrm{ix}}\phi_{\mathrm{xi}}\right)^2\left(c_{\mathrm{jj}}+\sigma_{\mathrm{jx}}\phi_{\mathrm{xj}}\right)}}{2\sqrt{\frac{\left(c_{\mathrm{ij}}+\sigma_{\mathrm{ix}}\phi_{\mathrm{xj}}\right)\left(c_{\mathrm{ji}}+\sigma_{\mathrm{jx}}\phi_{\mathrm{xi}}\right)}{\left(c_{\mathrm{ii}}+\sigma_{\mathrm{ix}}\phi_{\mathrm{xi}}\right)\left(c_{\mathrm{jj}}+\sigma_{\mathrm{jx}}\phi_{\mathrm{xj}}\right)}}} \tag{7}$$

$$\frac{\partial ND}{\partial \sigma_{jx}} = -\frac{\frac{\phi_{\mathrm{xi}}\left(c_{\mathrm{ij}}+\sigma_{\mathrm{ix}}\phi_{\mathrm{xj}}\right)}{\left(c_{\mathrm{ii}}+\sigma_{\mathrm{ix}}\phi_{\mathrm{xi}}\right)\left(c_{\mathrm{jj}}+\sigma_{\mathrm{jx}}\phi_{\mathrm{xj}}\right)} - \frac{\phi_{\mathrm{xj}}\left(c_{\mathrm{ij}}+\sigma_{\mathrm{ix}}\phi_{\mathrm{xj}}\right)\left(c_{\mathrm{ji}}+\sigma_{\mathrm{jx}}\phi_{\mathrm{xi}}\right)}{\left(c_{\mathrm{ii}}+\sigma_{\mathrm{ix}}\phi_{\mathrm{xi}}\right)\left(c_{\mathrm{jj}}+\sigma_{\mathrm{jx}}\phi_{\mathrm{xj}}\right)^2}}{2\sqrt{\frac{\left(c_{\mathrm{ij}}+\sigma_{\mathrm{ix}}\phi_{\mathrm{xj}}\right)\left(c_{\mathrm{ji}}+\sigma_{\mathrm{jx}}\phi_{\mathrm{xi}}\right)}{\left(c_{\mathrm{ii}}+\sigma_{\mathrm{ix}}\phi_{\mathrm{xi}}\right)\left(c_{\mathrm{jj}}+\sigma_{\mathrm{jx}}\phi_{\mathrm{xj}}\right)}}} \tag{8}$$

$$\frac{\partial ND}{\partial \phi_{xi}} = -\frac{\frac{\sigma_{\mathrm{jx}}\left(c_{\mathrm{ij}}+\sigma_{\mathrm{ix}}\phi_{\mathrm{xj}}\right)}{\left(c_{\mathrm{ii}}+\sigma_{\mathrm{ix}}\phi_{\mathrm{xi}}\right)\left(c_{\mathrm{jj}}+\sigma_{\mathrm{jx}}\phi_{\mathrm{xj}}\right)} - \frac{\sigma_{\mathrm{ix}}\left(c_{\mathrm{ij}}+\sigma_{\mathrm{ix}}\phi_{\mathrm{xj}}\right)\left(c_{\mathrm{ji}}+\sigma_{\mathrm{jx}}\phi_{\mathrm{xi}}\right)}{\left(c_{\mathrm{ii}}+\sigma_{\mathrm{ix}}\phi_{\mathrm{xi}}\right)^2\left(c_{\mathrm{jj}}+\sigma_{\mathrm{jx}}\phi_{\mathrm{xj}}\right)}}{2\sqrt{\frac{\left(c_{\mathrm{ij}}+\sigma_{\mathrm{ix}}\phi_{\mathrm{xj}}\right)\left(c_{\mathrm{ji}}+\sigma_{\mathrm{jx}}\phi_{\mathrm{xi}}\right)}{\left(c_{\mathrm{ii}}+\sigma_{\mathrm{ix}}\phi_{\mathrm{xi}}\right)\left(c_{\mathrm{jj}}+\sigma_{\mathrm{jx}}\phi_{\mathrm{xj}}\right)}}} \tag{9}$$

$$\frac{\partial ND}{\partial \phi_{xj}} = -\frac{\frac{\sigma_{\mathrm{ix}}\left(c_{\mathrm{ji}}+\sigma_{\mathrm{jx}}\phi_{\mathrm{xi}}\right)}{\left(c_{\mathrm{ii}}+\sigma_{\mathrm{ix}}\phi_{\mathrm{xi}}\right)\left(c_{\mathrm{jj}}+\sigma_{\mathrm{jx}}\phi_{\mathrm{xj}}\right)} - \frac{\sigma_{\mathrm{jx}}\left(c_{\mathrm{ij}}+\sigma_{\mathrm{ix}}\phi_{\mathrm{xj}}\right)\left(c_{\mathrm{ji}}+\sigma_{\mathrm{jx}}\phi_{\mathrm{xi}}\right)}{\left(c_{\mathrm{ii}}+\sigma_{\mathrm{ix}}\phi_{\mathrm{xi}}\right)\left(c_{\mathrm{jj}}+\sigma_{\mathrm{jx}}\phi_{\mathrm{xj}}\right)^2}}{2\sqrt{\frac{\left(c_{\mathrm{ij}}+\sigma_{\mathrm{ix}}\phi_{\mathrm{xj}}\right)\left(c_{\mathrm{ji}}+\sigma_{\mathrm{jx}}\phi_{\mathrm{xi}}\right)}{\left(c_{\mathrm{ii}}+\sigma_{\mathrm{ix}}\phi_{\mathrm{xi}}\right)\left(c_{\mathrm{jj}}+\sigma_{\mathrm{jx}}\phi_{\mathrm{xj}}\right)}}} \tag{10}$$

Fitness Ratio

$$\frac{\partial FR}{\partial c_{ij}} = \frac{c_{\text{ii}} + \sigma_{\text{ix}}\phi_{\text{xi}}}{2\left(c_{\text{ji}} + \sigma_{\text{jx}}\phi_{\text{xi}}\right)\left(c_{\text{jj}} + \sigma_{\text{jx}}\phi_{\text{xj}}\right)\sqrt{\frac{\left(c_{\text{ii}}+\sigma_{\text{ix}}\phi_{\text{xi}}\right)\left(c_{\text{ij}}+\sigma_{\text{ix}}\phi_{\text{xj}}\right)}{\left(c_{\text{ji}}+\sigma_{\text{jx}}\phi_{\text{xi}}\right)\left(c_{\text{jj}}+\sigma_{\text{jx}}\phi_{\text{xj}}\right)}}} \tag{11}$$

$$\frac{\partial FR}{\partial c_{ji}} = -\frac{\left(c_{\text{ii}} + \sigma_{\text{ix}}\phi_{\text{xi}}\right)\left(c_{\text{ij}} + \sigma_{\text{ix}}\phi_{\text{xj}}\right)}{2\left(c_{\text{ji}} + \sigma_{\text{jx}}\phi_{\text{xi}}\right)^2\left(c_{\text{jj}} + \sigma_{\text{jx}}\phi_{\text{xj}}\right)\sqrt{\frac{\left(c_{\text{ii}}+\sigma_{\text{ix}}\phi_{\text{xi}}\right)\left(c_{\text{ij}}+\sigma_{\text{ix}}\phi_{\text{xj}}\right)}{\left(c_{\text{ji}}+\sigma_{\text{jx}}\phi_{\text{xi}}\right)\left(c_{\text{jj}}+\sigma_{\text{jx}}\phi_{\text{xj}}\right)}}} \tag{12}$$

$$\frac{\partial FR}{\partial c_{ii}} = \frac{c_{\text{ij}} + \sigma_{\text{ix}}\phi_{\text{xj}}}{2\left(c_{\text{ji}} + \sigma_{\text{jx}}\phi_{\text{xi}}\right)\left(c_{\text{jj}} + \sigma_{\text{jx}}\phi_{\text{xj}}\right)\sqrt{\frac{\left(c_{\text{ii}}+\sigma_{\text{ix}}\phi_{\text{xi}}\right)\left(c_{\text{ij}}+\sigma_{\text{ix}}\phi_{\text{xj}}\right)}{\left(c_{\text{ji}}+\sigma_{\text{jx}}\phi_{\text{xi}}\right)\left(c_{\text{jj}}+\sigma_{\text{jx}}\phi_{\text{xj}}\right)}}} \tag{13}$$

$$\frac{\partial FR}{\partial c_{jj}} = -\frac{\left(c_{\text{ii}} + \sigma_{\text{ix}}\phi_{\text{xi}}\right)\left(c_{\text{ij}} + \sigma_{\text{ix}}\phi_{\text{xj}}\right)}{2\left(c_{\text{ji}} + \sigma_{\text{jx}}\phi_{\text{xi}}\right)\left(c_{\text{jj}} + \sigma_{\text{jx}}\phi_{\text{xj}}\right)^2\sqrt{\frac{\left(c_{\text{ii}}+\sigma_{\text{ix}}\phi_{\text{xi}}\right)\left(c_{\text{ij}}+\sigma_{\text{ix}}\phi_{\text{xj}}\right)}{\left(c_{\text{ji}}+\sigma_{\text{jx}}\phi_{\text{xi}}\right)\left(c_{\text{jj}}+\sigma_{\text{jx}}\phi_{\text{xj}}\right)}}} \tag{14}$$

$$\frac{\partial FR}{\partial \sigma_{ix}} = \frac{\frac{\phi_{\text{xj}}\left(c_{\text{ii}}+\sigma_{\text{ix}}\phi_{\text{xi}}\right)}{\left(c_{\text{ji}}+\sigma_{\text{jx}}\phi_{\text{xi}}\right)\left(c_{\text{jj}}+\sigma_{\text{jx}}\phi_{\text{xj}}\right)} + \frac{\phi_{\text{xi}}\left(c_{\text{ij}}+\sigma_{\text{ix}}\phi_{\text{xj}}\right)}{\left(c_{\text{ji}}+\sigma_{\text{jx}}\phi_{\text{xi}}\right)\left(c_{\text{jj}}+\sigma_{\text{jx}}\phi_{\text{xj}}\right)}}{2\sqrt{\frac{\left(c_{\text{ii}}+\sigma_{\text{ix}}\phi_{\text{xi}}\right)\left(c_{\text{ij}}+\sigma_{\text{ix}}\phi_{\text{xj}}\right)}{\left(c_{\text{ji}}+\sigma_{\text{jx}}\phi_{\text{xi}}\right)\left(c_{\text{jj}}+\sigma_{\text{jx}}\phi_{\text{xj}}\right)}}} \tag{15}$$

$$\frac{\partial FR}{\partial \sigma_{jx}} = \frac{-\frac{\phi_{\text{xi}}\left(c_{\text{ii}}+\sigma_{\text{ix}}\phi_{\text{xi}}\right)\left(c_{\text{ij}}+\sigma_{\text{ix}}\phi_{\text{xj}}\right)}{\left(c_{\text{ji}}+\sigma_{\text{jx}}\phi_{\text{xi}}\right)^2\left(c_{\text{jj}}+\sigma_{\text{jx}}\phi_{\text{xj}}\right)} - \frac{\phi_{\text{xj}}\left(c_{\text{ii}}+\sigma_{\text{ix}}\phi_{\text{xi}}\right)\left(c_{\text{ij}}+\sigma_{\text{ix}}\phi_{\text{xj}}\right)}{\left(c_{\text{ji}}+\sigma_{\text{jx}}\phi_{\text{xi}}\right)\left(c_{\text{jj}}+\sigma_{\text{jx}}\phi_{\text{xj}}\right)^2}}{2\sqrt{\frac{\left(c_{\text{ii}}+\sigma_{\text{ix}}\phi_{\text{xi}}\right)\left(c_{\text{ij}}+\sigma_{\text{ix}}\phi_{\text{xj}}\right)}{\left(c_{\text{ji}}+\sigma_{\text{jx}}\phi_{\text{xi}}\right)\left(c_{\text{jj}}+\sigma_{\text{jx}}\phi_{\text{xj}}\right)}}} \tag{16}$$

$$\frac{\partial FR}{\partial \phi_{xi}} = \frac{\frac{\sigma_{\text{ix}}\left(c_{\text{ij}}+\sigma_{\text{ix}}\phi_{\text{xj}}\right)}{\left(c_{\text{ji}}+\sigma_{\text{jx}}\phi_{\text{xi}}\right)\left(c_{\text{jj}}+\sigma_{\text{jx}}\phi_{\text{xj}}\right)} - \frac{\sigma_{\text{jx}}\left(c_{\text{ii}}+\sigma_{\text{ix}}\phi_{\text{xi}}\right)\left(c_{\text{ij}}+\sigma_{\text{ix}}\phi_{\text{xj}}\right)}{\left(c_{\text{ji}}+\sigma_{\text{jx}}\phi_{\text{xi}}\right)^2\left(c_{\text{jj}}+\sigma_{\text{jx}}\phi_{\text{xj}}\right)}}{2\sqrt{\frac{\left(c_{\text{ii}}+\sigma_{\text{ix}}\phi_{\text{xi}}\right)\left(c_{\text{ij}}+\sigma_{\text{ix}}\phi_{\text{xj}}\right)}{\left(c_{\text{ji}}+\sigma_{\text{jx}}\phi_{\text{xi}}\right)\left(c_{\text{jj}}+\sigma_{\text{jx}}\phi_{\text{xj}}\right)}}} \tag{17}$$

$$\frac{\partial FR}{\partial \phi_{xj}} = \frac{\frac{\sigma_{\text{ix}}\left(c_{\text{ii}}+\sigma_{\text{ix}}\phi_{\text{xi}}\right)}{\left(c_{\text{ji}}+\sigma_{\text{jx}}\phi_{\text{xi}}\right)\left(c_{\text{jj}}+\sigma_{\text{jx}}\phi_{\text{xj}}\right)} - \frac{\sigma_{\text{jx}}\left(c_{\text{ii}}+\sigma_{\text{ix}}\phi_{\text{xi}}\right)\left(c_{\text{ij}}+\sigma_{\text{ix}}\phi_{\text{xj}}\right)}{\left(c_{\text{ji}}+\sigma_{\text{jx}}\phi_{\text{xi}}\right)\left(c_{\text{jj}}+\sigma_{\text{jx}}\phi_{\text{xj}}\right)^2}}{2\sqrt{\frac{\left(c_{\text{ii}}+\sigma_{\text{ix}}\phi_{\text{xi}}\right)\left(c_{\text{ij}}+\sigma_{\text{ix}}\phi_{\text{xj}}\right)}{\left(c_{\text{ji}}+\sigma_{\text{jx}}\phi_{\text{xi}}\right)\left(c_{\text{jj}}+\sigma_{\text{jx}}\phi_{\text{xj}}\right)}}} \tag{18}$$

2.1.2 m Parameterization

Niche Differences

$$\frac{\partial ND}{\partial c_{ij}} = -\frac{c_{\mathrm{ji}} + m_{\mathrm{ji}}}{2\left(c_{\mathrm{ii}} + m_{\mathrm{ii}}\right)\left(c_{\mathrm{jj}} + m_{\mathrm{jj}}\right)\sqrt{\frac{\left(c_{\mathrm{ij}}+m_{\mathrm{ij}}\right)\left(c_{\mathrm{ji}}+m_{\mathrm{ji}}\right)}{\left(c_{\mathrm{ii}}+m_{\mathrm{ii}}\right)\left(c_{\mathrm{jj}}+m_{\mathrm{jj}}\right)}}} \tag{19}$$

$$\frac{\partial ND}{\partial c_{ji}} = -\frac{c_{\mathrm{ij}} + m_{\mathrm{ij}}}{2\left(c_{\mathrm{ii}} + m_{\mathrm{ii}}\right)\left(c_{\mathrm{jj}} + m_{\mathrm{jj}}\right)\sqrt{\frac{\left(c_{\mathrm{ij}}+m_{\mathrm{ij}}\right)\left(c_{\mathrm{ji}}+m_{\mathrm{ji}}\right)}{\left(c_{\mathrm{ii}}+m_{\mathrm{ii}}\right)\left(c_{\mathrm{jj}}+m_{\mathrm{jj}}\right)}}} \tag{20}$$

$$\frac{\partial ND}{\partial c_{ii}} = \frac{\left(c_{\mathrm{ij}} + m_{\mathrm{ij}}\right)\left(c_{\mathrm{ji}} + m_{\mathrm{ji}}\right)}{2\left(c_{\mathrm{ii}} + m_{\mathrm{ii}}\right)^2\left(c_{\mathrm{jj}} + m_{\mathrm{jj}}\right)\sqrt{\frac{\left(c_{\mathrm{ij}}+m_{\mathrm{ij}}\right)\left(c_{\mathrm{ji}}+m_{\mathrm{ji}}\right)}{\left(c_{\mathrm{ii}}+m_{\mathrm{ii}}\right)\left(c_{\mathrm{jj}}+m_{\mathrm{jj}}\right)}}} \tag{21}$$

$$\frac{\partial ND}{\partial c_{jj}} = \frac{\left(c_{\mathrm{ij}} + m_{\mathrm{ij}}\right)\left(c_{\mathrm{ji}} + m_{\mathrm{ji}}\right)}{2\left(c_{\mathrm{ii}} + m_{\mathrm{ii}}\right)\left(c_{\mathrm{jj}} + m_{\mathrm{jj}}\right)^2\sqrt{\frac{\left(c_{\mathrm{ij}}+m_{\mathrm{ij}}\right)\left(c_{\mathrm{ji}}+m_{\mathrm{ji}}\right)}{\left(c_{\mathrm{ii}}+m_{\mathrm{ii}}\right)\left(c_{\mathrm{jj}}+m_{\mathrm{jj}}\right)}}} \tag{22}$$

$$\frac{\partial ND}{\partial m_{ij}} = -\frac{c_{\mathrm{ji}} + m_{\mathrm{ji}}}{2\left(c_{\mathrm{ii}} + m_{\mathrm{ii}}\right)\left(c_{\mathrm{jj}} + m_{\mathrm{jj}}\right)\sqrt{\frac{\left(c_{\mathrm{ij}}+m_{\mathrm{ij}}\right)\left(c_{\mathrm{ji}}+m_{\mathrm{ji}}\right)}{\left(c_{\mathrm{ii}}+m_{\mathrm{ii}}\right)\left(c_{\mathrm{jj}}+m_{\mathrm{jj}}\right)}}} \tag{23}$$

$$\frac{\partial ND}{\partial m_{ji}} = -\frac{c_{\mathrm{ij}} + m_{\mathrm{ij}}}{2\left(c_{\mathrm{ii}} + m_{\mathrm{ii}}\right)\left(c_{\mathrm{jj}} + m_{\mathrm{jj}}\right)\sqrt{\frac{\left(c_{\mathrm{ij}}+m_{\mathrm{ij}}\right)\left(c_{\mathrm{ji}}+m_{\mathrm{ji}}\right)}{\left(c_{\mathrm{ii}}+m_{\mathrm{ii}}\right)\left(c_{\mathrm{jj}}+m_{\mathrm{jj}}\right)}}} \tag{24}$$

$$\frac{\partial ND}{\partial m_{ii}} = \frac{\left(c_{\mathrm{ij}} + m_{\mathrm{ij}}\right)\left(c_{\mathrm{ji}} + m_{\mathrm{ji}}\right)}{2\left(c_{\mathrm{ii}} + m_{\mathrm{ii}}\right)^2\left(c_{\mathrm{jj}} + m_{\mathrm{jj}}\right)\sqrt{\frac{\left(c_{\mathrm{ij}}+m_{\mathrm{ij}}\right)\left(c_{\mathrm{ji}}+m_{\mathrm{ji}}\right)}{\left(c_{\mathrm{ii}}+m_{\mathrm{ii}}\right)\left(c_{\mathrm{jj}}+m_{\mathrm{jj}}\right)}}} \tag{25}$$

$$\frac{\partial ND}{\partial m_{jj}} = \frac{\left(c_{\mathrm{ij}} + m_{\mathrm{ij}}\right)\left(c_{\mathrm{ji}} + m_{\mathrm{ji}}\right)}{2\left(c_{\mathrm{ii}} + m_{\mathrm{ii}}\right)\left(c_{\mathrm{jj}} + m_{\mathrm{jj}}\right)^2\sqrt{\frac{\left(c_{\mathrm{ij}}+m_{\mathrm{ij}}\right)\left(c_{\mathrm{ji}}+m_{\mathrm{ji}}\right)}{\left(c_{\mathrm{ii}}+m_{\mathrm{ii}}\right)\left(c_{\mathrm{jj}}+m_{\mathrm{jj}}\right)}}} \tag{26}$$

Fitness Ratio

$$\frac{\partial FR}{\partial c_{ij}} = \frac{c_{\mathrm{ii}} + m_{\mathrm{ii}}}{2\left(c_{\mathrm{ji}} + m_{\mathrm{ji}}\right)\left(c_{\mathrm{jj}} + m_{\mathrm{jj}}\right)\sqrt{\frac{\left(c_{\mathrm{ii}}+m_{\mathrm{ii}}\right)\left(c_{\mathrm{ij}}+m_{\mathrm{ij}}\right)}{\left(c_{\mathrm{ji}}+m_{\mathrm{ji}}\right)\left(c_{\mathrm{jj}}+m_{\mathrm{jj}}\right)}}} \tag{27}$$

$$\frac{\partial FR}{\partial c_{ji}} = -\frac{\left(c_{\mathrm{ii}} + m_{\mathrm{ii}}\right)\left(c_{\mathrm{ij}} + m_{\mathrm{ij}}\right)}{2\left(c_{\mathrm{ji}} + m_{\mathrm{ji}}\right)^{2}\left(c_{\mathrm{jj}} + m_{\mathrm{jj}}\right)\sqrt{\frac{\left(c_{\mathrm{ii}}+m_{\mathrm{ii}}\right)\left(c_{\mathrm{ij}}+m_{\mathrm{ij}}\right)}{\left(c_{\mathrm{ji}}+m_{\mathrm{ji}}\right)\left(c_{\mathrm{jj}}+m_{\mathrm{jj}}\right)}}} \tag{28}$$

$$\frac{\partial FR}{\partial c_{ii}} = \frac{c_{\mathrm{ij}} + m_{\mathrm{ij}}}{2\left(c_{\mathrm{ji}} + m_{\mathrm{ji}}\right)\left(c_{\mathrm{jj}} + m_{\mathrm{jj}}\right)\sqrt{\frac{\left(c_{\mathrm{ii}}+m_{\mathrm{ii}}\right)\left(c_{\mathrm{ij}}+m_{\mathrm{ij}}\right)}{\left(c_{\mathrm{ji}}+m_{\mathrm{ji}}\right)\left(c_{\mathrm{jj}}+m_{\mathrm{jj}}\right)}}} \tag{29}$$

$$\frac{\partial FR}{\partial c_{jj}} = -\frac{\left(c_{\mathrm{ii}} + m_{\mathrm{ii}}\right)\left(c_{\mathrm{ij}} + m_{\mathrm{ij}}\right)}{2\left(c_{\mathrm{ji}} + m_{\mathrm{ji}}\right)\left(c_{\mathrm{jj}} + m_{\mathrm{jj}}\right)^{2}\sqrt{\frac{\left(c_{\mathrm{ii}}+m_{\mathrm{ii}}\right)\left(c_{\mathrm{ij}}+m_{\mathrm{ij}}\right)}{\left(c_{\mathrm{ji}}+m_{\mathrm{ji}}\right)\left(c_{\mathrm{jj}}+m_{\mathrm{jj}}\right)}}} \tag{30}$$

$$\frac{\partial FR}{\partial m_{ij}} = \frac{c_{\mathrm{ii}} + m_{\mathrm{ii}}}{2\left(c_{\mathrm{ji}} + m_{\mathrm{ji}}\right)\left(c_{\mathrm{jj}} + m_{\mathrm{jj}}\right)\sqrt{\frac{\left(c_{\mathrm{ii}}+m_{\mathrm{ii}}\right)\left(c_{\mathrm{ij}}+m_{\mathrm{ij}}\right)}{\left(c_{\mathrm{ji}}+m_{\mathrm{ji}}\right)\left(c_{\mathrm{jj}}+m_{\mathrm{jj}}\right)}}} \tag{31}$$

$$\frac{\partial FR}{\partial m_{ji}} = -\frac{\left(c_{\mathrm{ii}} + m_{\mathrm{ii}}\right)\left(c_{\mathrm{ij}} + m_{\mathrm{ij}}\right)}{2\left(c_{\mathrm{ji}} + m_{\mathrm{ji}}\right)^{2}\left(c_{\mathrm{jj}} + m_{\mathrm{jj}}\right)\sqrt{\frac{\left(c_{\mathrm{ii}}+m_{\mathrm{ii}}\right)\left(c_{\mathrm{ij}}+m_{\mathrm{ij}}\right)}{\left(c_{\mathrm{ji}}+m_{\mathrm{ji}}\right)\left(c_{\mathrm{jj}}+m_{\mathrm{jj}}\right)}}} \tag{32}$$

$$\frac{\partial FR}{\partial m_{ii}} = \frac{c_{\mathrm{ij}} + m_{\mathrm{ij}}}{2\left(c_{\mathrm{ji}} + m_{\mathrm{ji}}\right)\left(c_{\mathrm{jj}} + m_{\mathrm{jj}}\right)\sqrt{\frac{\left(c_{\mathrm{ii}}+m_{\mathrm{ii}}\right)\left(c_{\mathrm{ij}}+m_{\mathrm{ij}}\right)}{\left(c_{\mathrm{ji}}+m_{\mathrm{ji}}\right)\left(c_{\mathrm{jj}}+m_{\mathrm{jj}}\right)}}} \tag{33}$$

$$\frac{\partial FR}{\partial m_{jj}} = -\frac{\left(c_{\mathrm{ii}} + m_{\mathrm{ii}}\right)\left(c_{\mathrm{ij}} + m_{\mathrm{ij}}\right)}{2\left(c_{\mathrm{ji}} + m_{\mathrm{ji}}\right)\left(c_{\mathrm{jj}} + m_{\mathrm{jj}}\right)^{2}\sqrt{\frac{\left(c_{\mathrm{ii}}+m_{\mathrm{ii}}\right)\left(c_{\mathrm{ij}}+m_{\mathrm{ij}}\right)}{\left(c_{\mathrm{ji}}+m_{\mathrm{ji}}\right)\left(c_{\mathrm{jj}}+m_{\mathrm{jj}}\right)}}} \tag{34}$$

3 PRCC Based Sensitivity Analysis

Finally, we conducted a partial-rank correlation coefficient (PRCC) sensitivity analysis drawing 100,000 combinations of parameter estimates from Uniform distributions and estimating the PRCC between each parameter and either the niche differences or fitness ratios (Wu *et al.* 2013; Figure S4). This analysis allows all parameters to vary uniformly at random across their given distributions; thus results are not dependent on all other parameters being fixed, such as occurs in the local sensitivity analysis. The minimun and maximum parameter values were the same as those for the local sensitivity analysis: $c_{ij}[-0.1, 0]$; cultivation rates $\phi_{xi}[0, 20]$; microbial effects on plants $\sigma_{ix}[-0.01, 0.01]$; and microbial feedbacks $m_{ij}[-.2, .2]$. The PRCC was estimated using epiR version 2.0.7 (Stevenson *et al.*, 2024). [This is where I will put the results of this analysis... they confuse me though...]

4 Role of Taxonomic Richness

To assess whether the taxonomic richness of the microbial system impacts the results of the sensitivity analysis, we repeated the analytical local sensitivity analysis three times, sequentially adding an additional pathogen taxon. All microbial taxa in this set of analyses were complete generalists, being cultivated by and affecting both plant species. We assumed microbial taxa had similar but unidentical interactions with each plant, so each additional pathogen was slightly more pathogenic and had a slightly higher cultivation rate by both species. Thus, in addition to the baseline parameterization used in the one species model, we used the following additional parameter for species interactions involving microbial taxa y and z: $\phi_{yi} = 10.6$, $\phi_{yj} = 10.1$, $\sigma_{iy} = -0.0021$, $\sigma_{jy} = -0.00235$, $\phi_{zi} = 10.7$, $\phi_{zj} = 10.2$, $\sigma_{iz} = -0.0022$, and $\sigma_{jz} = -0.00245$. We also ran these sensitivity analyses scaling the cultivation rates by species richness such that ϕ was the ratio of the values above and the number of microbial taxa in the system.

The key finding that plant-microbe interactions alter fitness ratios more so than niche differences, whereas plant-plant interactions seem to alter fitness ratios and niche differences similarly persists across the three models. The main difference across these three models was the relative magnitude sensitivity of fitness ratios and niche differences between plant-plant terms and plant-microbe terms. For the models in which we did not scale the cultivation rates by taxonomic richness, the sensitivity of these terms to plant-microbe interactions increased with taxonomic richness at a much faster rate than the that of the plant-plant terms. Conversely, in the models where we scaled the cultivation rate by taxonomic richness, the relative differences between plant-plant interactions and plant-microbe interactions stayed the same across taxonomic richness.

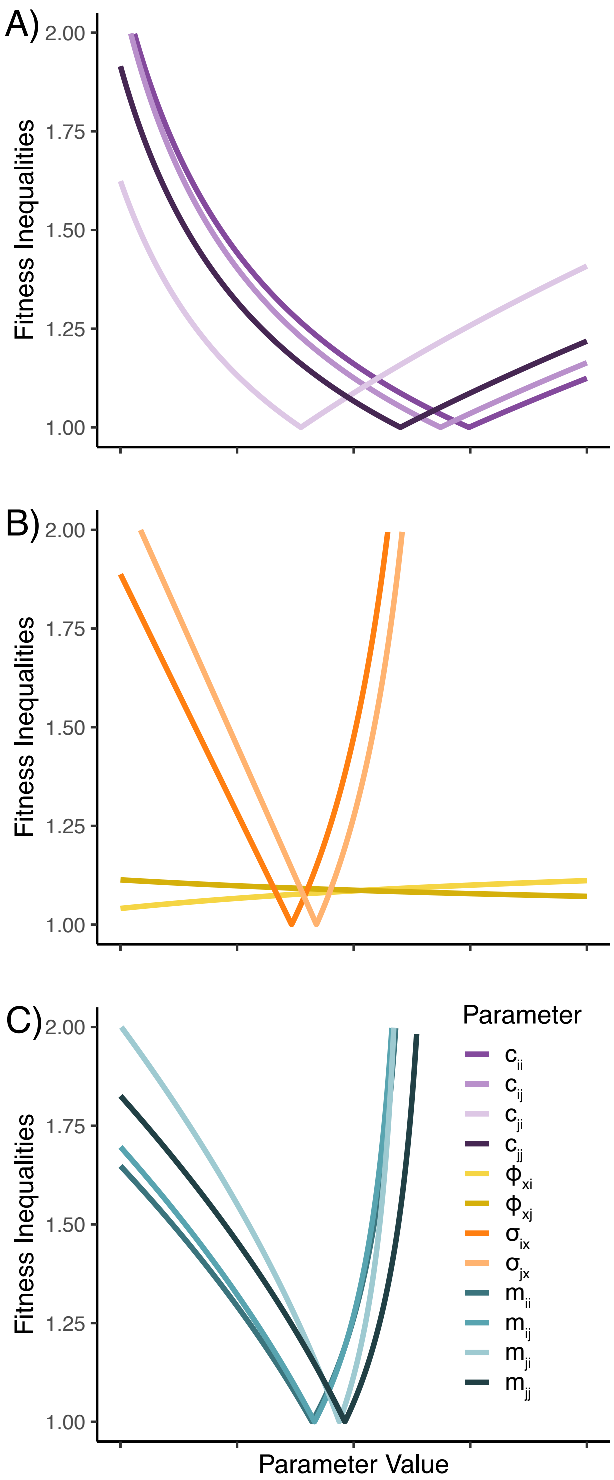

Figure S1: Local sensitivity analysis of the taxon specific plant-soil feedback Lotka-Volterra model. Lines represent the changes in fitness inequalities as the focal parameter varies from it's minimum to maximum value while all other parameters are held constant at their baseline values. Panels A, B, and C represent sensitivities to microbe-independent competition, plant-microbe interactions, and microbial feedbacks respectively

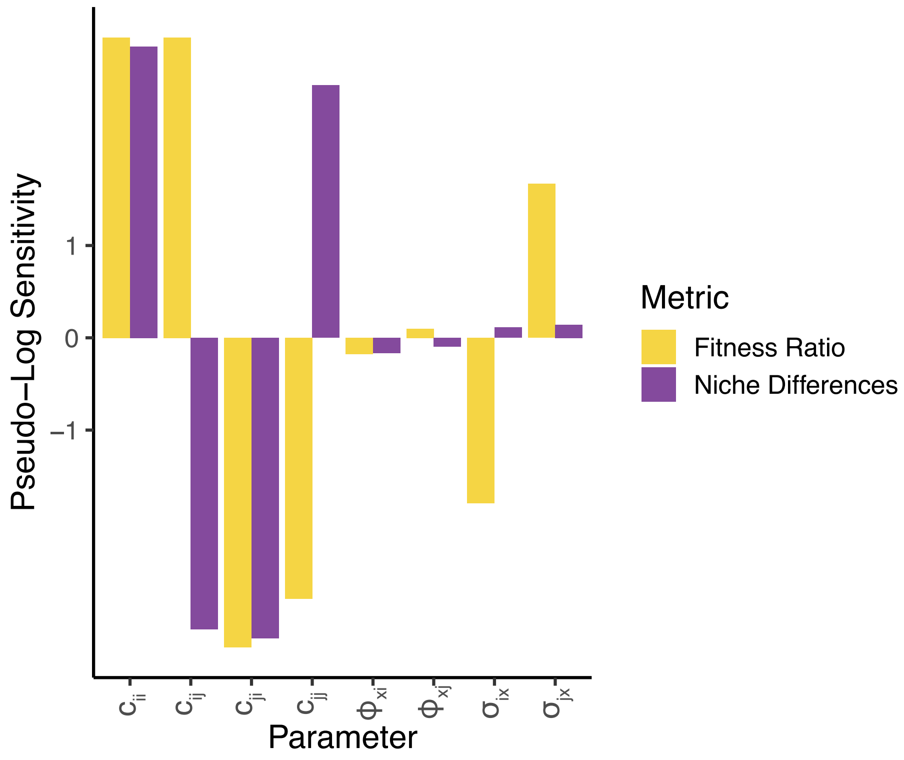

Figure S2: Comparison of microbe-independent plant competition and plant-microbe interaction term partial derivatives for the fitness ratio and niche differences calculated at the baseline parameter values.

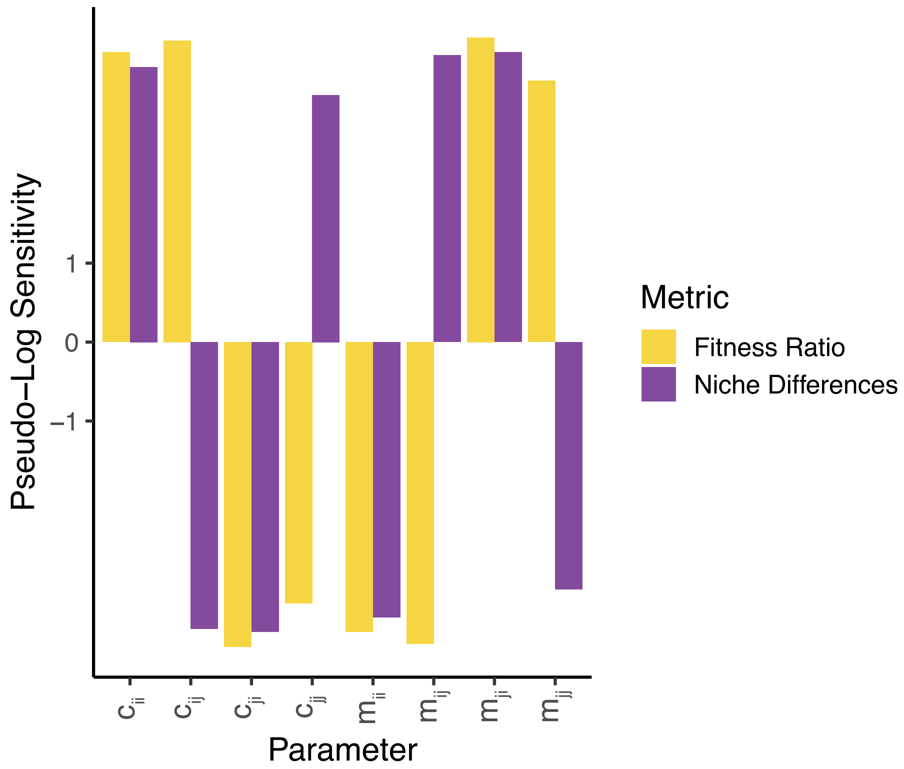

Figure S3: Comparison of microbe-independent plant competition and microbial feedback term partial derivatives for the fitness ratio and niche differences calculated at the baseline parameter values.

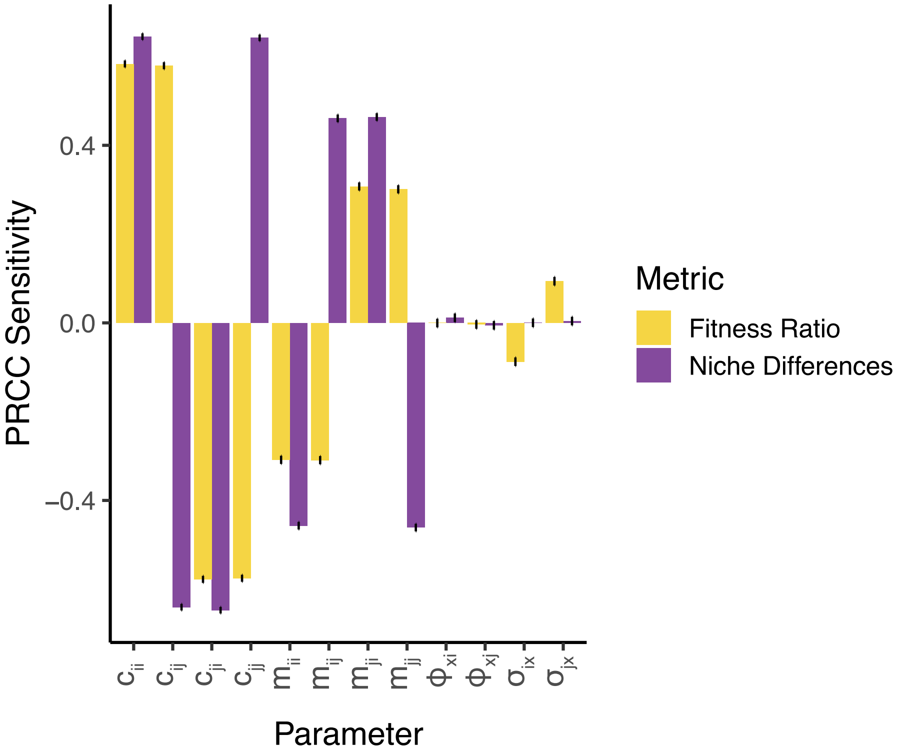

Figure S4: PRCC estimates for each parameter for the fitness ratio and niche differences. Error bars represent the 95% confidence intervals.

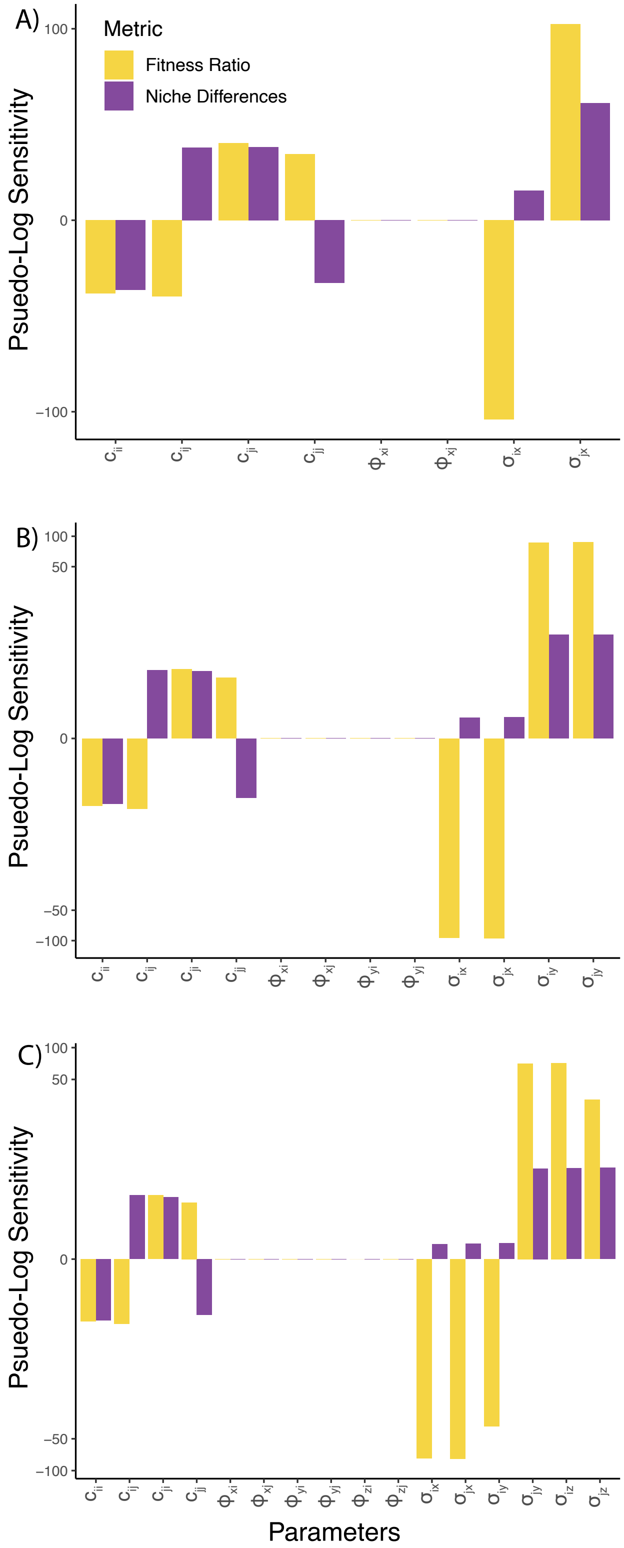

Figure S5: Comparison of microbe-independent plant competition and plant-microbe interaction term partial derivatives for the fitness ratio and niche differences calculated at the baseline parameter values across one (A), two (B), and three (C) microbe systems. These values are from the model run with unscaled cultivation rates.

References

Stevenson, M., Sergeant, E. & Firestone, S. (2024). *epiR: Tools for the Analysis of Epidemiological Data*. URL `https://CRAN.R-project.org/package=epiR`. R package version 2.0.70.

Wu, J., Dhingra, R., Gambhir, M. & Remais, J. V. (2013). Sensitivity analysis of infectious disease models: methods, advances and their application. *Journal of The Royal Society Interface*, 10, 20121018.
