# Supplementary material for: Environmental context alters plant–soil feedback effects on plant coexistence
Source: Ecology. 2025 Aug 6;106(8):e70170. doi: 10.1002/ecy.70170 (PMC12327179; doi:10.1002/ecy.70170)
Supplement: Supplementary file 4 — Appendix S4. [file ECY-106-e70170-s004.pdf]

Appendix S4: Model Structure and Parameterization of Invasion Simulations

Environmental context alters plant-soil feedback effects on plant coexistence

Jeremy A. Collings, Lauren G. Shoemaker & Jeffrey M. Diez

in *Ecology*

1 Species Specific Pathogen Model Structure

In our first set of simulations considering the invasive plant, *Alliaria petiolata* and containing only a species specific pathogen associated with the competitively dominant plant species, plant population growth was modeled as:

$$\frac{dN_A}{dt} = r_A N_A (1 + c_{AA} N_A + c_{AB} N_B + \sigma_{AX} S_X) \tag{1}$$

$$\frac{dN_B}{dt} = r_B N_B (1 + c_{BB} N_B + c_{BA} N_A) \tag{2}$$

The population growth of the pathogenic microbial taxon was modeled as:

$$\frac{dS_X}{dt} = g_X S_X (1 - \frac{S_X}{k_X}) \tag{3}$$

where the carrying capacity is modeled as:

$$k_X = \phi_{XA} N_A \tag{4}$$

Thus, applying a separation of timescales between plant and microbe population dynamics as performed by Ke and Wan (2020), we can calculate competition coefficients by assuming that the microbial taxon is always at carrying capacity and substituting this into the plant population growth models such that, for example, the population growth rate of *Alliaria petiolata* can be rewritten as

$$\frac{dN_A}{dt} = r_A N_A (1 + (c_{AA} + \sigma_{AX} \phi_{XA}) N_A + c_{AB} N_B) \tag{5}$$

Thus, our competition coefficients for our models including only the species specific pathogen are:

$$\alpha_{AA} = c_{AA} + \sigma_{AX}\phi_{XA} \tag{6}$$

$$\alpha_{BA} = c_{BA} \tag{7}$$

$$\alpha_{BB} = c_{BB} \tag{8}$$

$$\alpha_{AB} = c_{AB} \tag{9}$$

2 Species Specific Mutualist Model Structure

Our next set of simulations included a species specific mutualist (Y) for the native competitor such that the population growth rate for *Alliaria petiolata* stayed the same, but the growth rate for the native competitor was modeled as:

$$\frac{dN_B}{dt} = r_B N_B(1 + c_{BB}N_B + c_{BA}N_A + \sigma_{BY}) \tag{10}$$

The incorporation of a mutualist that is both cultivated by and influencing the growth rate of the native competitor alters intraspecific competition for the native species such that:

$$\alpha_{BB} = c_{BB} + \sigma_{BY}\phi_{YB} \tag{11}$$

while all other competition coefficients are calculated similarly to the previous set of simulations.

3 Generalist Decomposer Model Structure

Our final set of simulations included a generalist decomposer (Z) that interacted with both *Alliaria petiolata* and the native competitor such that their population growth rates were modeled as:

$$\frac{dN_A}{dt} = r_A N_A(1 + c_{AA}N_A + c_{AB}N_B + \sigma_{AX}S_X + \sigma_{AZ}S_Z) \tag{12}$$

$$\frac{dN_B}{dt} = r_B N_B(1 + c_{BB}N_B + c_{BA}N_A + \sigma_{BY}S_Y + \sigma_{BZ}S_Z) \tag{13}$$

Incorporating a generalist microbial taxon alters all competition coefficients such that now, competition coefficients can be calculated as:

$$\alpha_{AA} = c_{AA} + \sigma_{AX}\phi_{XA} + \sigma_{AZ}\phi_{ZA} \tag{14}$$

$$\alpha_{BA} = c_{BA} + \sigma_{BZ}\phi_{ZA} \quad (15)$$

$$\alpha_{BB} = c_{BB} + \sigma_{BY}\phi_{YB} + \sigma_{BZ}\phi_{ZB} \quad (16)$$

$$\alpha_{AB} = c_{AB} + \sigma_{AZ}\phi_{ZB} \quad (17)$$

4 Parameterization

To start, we set the microbe-independent plant competition parameters such that *Alliaria petiolata* would competitively exclude the native competitor under sterile conditions where $c_{AA} = -0.005$, $c_{BA} = -0.008$, $c_{BB} = -0.008$, and $c_{AB} = -0.oo5$. Because niche and fitness differences are calculated with only the overall competition coefficients (α_{ij}), we did not define parameter values for the plant growth rates (r_i). To incorporate microbial effects, we set the baseline per capita effect of the pathogen on *A. petiolata* as $\hat{\sigma}_{0,AX} = -0.002$ and the baseline cultivation rate of the pathogen by *A. petiolata* as $\hat{\phi}_{0,XA} = 0.2$. Finally, we scaled the cultivation rate of the pathogen by rainfall (v) such that the pathogen was cultivated more quickly with increased rainfall: $\phi_{XA} = 0.2 + v$.

When incorporating the mutualist, we kept the parameterization above and added new baseline $\hat{\sigma}_{0,iX}$ and $\hat{\phi}_{0,Xi}$ parameters where $\hat{\sigma}_{0,BY} = 0.002$ and $\hat{\phi}_{0,YB} = 0.2$. We then scaled ϕ_{YB} such that the cultivation rate of the mutualist decreased with increased rainfall: $\phi_{YB} = 0.2 - 0.5 * v$.

Finally, when incorporating the generalist decomposer, we added new baseline parameters where $\hat{\sigma}_{0,AZ} = 0.003$, $\hat{\phi}_{0,ZA} = 0.2$, $\hat{\sigma}_{0,BZ} = 0.001$, and $\hat{\phi}_{0,ZB} = 0.2$. We then scaled the cultivation of the decomposer by both plant species such that cultivation rates increased similarly with increased precipitation: $\phi_{ZA} = 0.2 + v$ and $\phi_{ZB} = 0.2 + v$.

For each simulation, we calculated competition coefficients as defined above and niche differences and fitness ratios as defined in the main text. The finding of Lozano et al. (2021) informed our decisions about how to scale the cultivation rates of each fungal guild in response to the precipitation gradient.

References

Ke, P.-J. & Wan, J. (2020). Effects of soil microbes on plant competition: A perspective from modern coexistence theory. *Ecological Monographs*, 90, e01391.

Lozano, Y. M., Aguilar-Trigueros, C. A., Roy, J. & Rillig, M. C. (2021). Drought induces shifts in soil fungal communities that can be linked to root traits across 24 plant species. *New Phytologist*, 232, 1917–1929.
